# Supplementary material for: Efficacy and moderators of psychological interventions in treating subclinical symptoms of depression and preventing major depressive disorder onsets: protocol for an individual patient data meta-analysis of randomised controlled trials
Source: BMJ Open. 2018 Mar 16;8(3):e018582. doi: 10.1136/bmjopen-2017-018582 (PMC5857689; doi:10.1136/bmjopen-2017-018582)
Supplement: Author's manuscript [file bmjopen-2017-018582.draft_revisions.pdf]

BMJ Open is committed to open peer review. As part of this commitment we make the peer review history of every article we publish publicly available.

When an article is published we post the peer reviewers' comments and the authors' responses online. We also post the versions of the paper that were used during peer review. These are the versions that the peer review comments apply to.

The versions of the paper that follow are the versions that were submitted during the peer review process. They are not the versions of record or the final published versions. They should not be cited or distributed as the published version of this manuscript.

BMJ Open is an open access journal and the full, final, typeset and author-corrected version of record of the manuscript is available on our site with no access controls, subscription charges or pay-per-view fees (http://bmjopen.bmj.com).

If you have any questions on BMJ Open's open peer review process please email editorial.bmjopen@bmj.com

BMJ Open

Efficacy and moderators of psychological interventions in treating subclinical symptoms of depression and preventing Major Depressive Disorder. Protocol for an individual patient data meta-analysis of randomized controlled trials

Journal:	*BMJ Open*
Manuscript ID	bmjopen-2017-018582
Article Type:	Protocol
Date Submitted by the Author:	18-Jul-2017

Complete List of Authors:	Ebert, David; Friedrich-Alexander University Erlangen Nuremberg, Clinical Psychology and Psychotherapy<br>Buntrock, Claudia; Friedrich-Alexander-Universitat Erlangen-Nurnberg, Clinical Psychology and Psychotherapy<br>Reins, Jo Annika; Leuphana Universitat Luneburg<br>Zimmermann, Johannes; Psychologische Hochschule Berlin<br>Cuijpers, Pim; VU University Amsterdam, Department of Clinical Psychology

Keywords:	MENTAL HEALTH, Depression & mood disorders < PSYCHIATRY, PREVENTIVE MEDICINE

SCHOLARONE™
Manuscripts

For peer review only

Efficacy and moderators of psychological interventions in treating subclinical symptoms of depression and preventing Major Depressive Disorder. Protocol for an individual patient data meta-analysis of randomized controlled trials

David Daniel Ebert[1]*, Claudia Buntrock[1], Jo Annika Reins[2], Johannes Zimmermann[3], Pim Cuijpers[4]

[1] Department of Clinical Psychology and Psychotherapy, Friedrich-Alexander-University Erlangen-Nuremberg, Naegelsbachstraße 25a, 91052 Erlangen, Germany

[2] Institute of Psychology, Leuphana University Lueneburg, Scharnhorststraße 1, 21335 Lueneburg, Germany

[3] Psychologische Hochschule Berlin, Am Köllnischen Park 2, 10179 Berlin, Germany

[4] Department of Clinical, Neuro and Developmental Psychology, EMGO+ Institute for Health and Care Research, VU University Amsterdam, Van der Boechorststraat 1, 1081 BT, Amsterdam, The Netherlands

*Corresponding author:

Dr. David Daniel Ebert
Friedrich-Alexander-University Erlangen-Nürnberg,
Department of Clinical Psychology and Psychotherapy
Naegelsbachstr. 25a, 91052 Erlangen, Germany
david.ebert@fau.de

For peer review only

Abstract

Introduction

The long-term effectiveness of psychological interventions for subclinical depression and the prevention of depression is unclear and effects vary among subgroups of patients indicating that not all patients profit from such interventions. Randomized trials are mostly underpowered to adequately examine subgroups and moderator effects. The aim of the present study is, therefore, to examine the short and long-term effects of psychological interventions compared to control groups in adults with subthreshold depression on depression symptom severity, treatment response, remission, deterioration, quality of life, anxiety, and the prevention of MDD onsets and moderators on individual patient- and study level using an individual-patient data meta-analysis approach.

Methods and analysis

Systematic searches in PubMed, PsycINFO, Embase and the Cochrane Central Register of Controlled Trials (CENTRAL) have been conducted. We will use the following types of outcome criteria: a) incidence of major depression, b) time to major depression onset, c) observer- and self-reported depression severity, d) response, e) remission, f) deterioration, g) quality of life, and h) anxiety. Multilevel models with participants nested within studies will be used. Missing data will be handled using a joint modeling approach to multiple imputation. A number of sensitivity analyses will be conducted in order test the robustness of our findings.

Ethics and dissemination

The original investigators have obtained ethical approval for the data used in the present study. This study will summarize the available evidence on the short- and long-term effectiveness of preventive psychological interventions for the treatment of subthreshold depression and prevention of major depressive disorder. Identification of subgroups of patients in which those interventions are most effective will guide the development of evidence-based personalized interventions for patients with subthreshold depression.

Systematic review registration

This study has been registered with the PROSPERO database (no. CRD42017058585).

For peer review only

Strengths and limitations of this study

- A strength of the presented individual patient-data meta-analysis (IPD MA) is that this approach allows sufficient statistical power to evaluate specific effects for specific kinds of treatments for patients with certain characteristics, in order to select the best possible treatment for an individual patient (i.e. personalized medicine).
- One limitation of the IPD MA is that while investigating moderators of treatment outcome, one very much relies on the variables that have been assessed in the primary studies. However, many of the relevant predictors and moderators associated with depression onset or differential treatment response reported in the literature were not assessed in the included studies.
- Another limitation of the IPD MA approach is that some bias is introduced because not all eligible trials can be included in the analyses due to author non-response, lack of ethical approval to share the data or that data are not available anymore.

For peer review only

Introduction

Major Depressive Disorder (MDD) is highly prevalent,[1–4] associated with substantial impairment[5,6] and economic costs.[7–9] Psychological treatments have been shown to be effective in the treatment of depression.[10,11] However, it has been estimated that, even when assuming the hypothetical scenario of full coverage with and adherence to evidence-based treatments only approximately one third of the disease burden attributable to MDD can be averted.[12] Moreover, in practice the majority of depressed people remain untreated[3,13] even in high income countries.[14,15]

Therefore, attention has increasingly been focused on the prevention of MDD.[16,17] One specific form of prevention is indicated prevention. In such interventions subthreshold symptoms are treated in order to prevent the transition to a full-blown depressive disorder.[17]

Meta-analytic evidence shows that indicated psychological preventive approaches can be effective in preventing depressive episodes.[18] The latest systematic review on this topic, which included randomized trials that have been published up to March 2012, found psychological interventions for subclinical symptoms to reduce the risk for developing a Major Depressive Episode at 6 months (Incidence rate ratio[IRR] = .61; 5 studies)) and at 12 months (IRR = .74; 4 studies). Since then, many more randomized controlled trials have been published, warranting an update of the evidence.

Moreover, the treatment of subclinical symptoms of depression itself is relevant. Subthreshold depressive symptoms are highly prevalent,[19] related to increased mortality,[20] poorer quality of life,[21] increased health care service utilisation,[22] and vast economic costs.[23] However, results for the treatment of subclinical symptoms are yet conflicting. Pharmacological interventions are unlikely to have a clinical advantage over placebos in treating subthreshold depression.[24] A recent meta-analysis, however, found small-to-moderate effect sizes for psychological interventions on depressive symptom severity at post-treatment compared to usual care.[25] However, four studies using clinician-rated outcomes did not indicate significant positive results. Moreover, we are not aware of any systematic review exploring the long-term effects of treatments for subclinical symptoms with regard to depression severity. In addition, effects on other relevant outcomes such as anxiety or quality of life have not been examined.

Another issue not yet addressed is the possibility that the effectiveness of psychological interventions for subthreshold depression varies across patients and not all subgroups of patients profit from such interventions. Given that the number of people from specific subgroups is often small in single trials, and randomized trials are usually powered to detect overall treatment effects,

For peer review only

RCTs are mostly underpowered to adequately examine subgroup and moderator analyses.[26] As studies also seldom report effectiveness for different patient characteristics, it is impossible to examine patient-level moderators using traditional meta-analytic approaches.

Individual participant data meta-analyses (IPD MA) can overcome some of the limitations of the conventional MAs on study level.[27–29] By pooling the primary data of individual trials, it is possible to conduct analyses not reported in original studies and obtain large enough sample sizes with sufficient power to examine effects in relevant subgroups and identify outcome moderators.[30]

The present study aims to examine the short and long-term effects of psychological interventions compared to control groups in adults with subthreshold depression on depression symptom severity, treatment response, remission, deterioration, quality of life, anxiety, and the prevention of MDD onsets using an IPD MA approach. Moderators on individual patient-level (e.g., socio-demographic, clinical characteristics) and study level (e.g., type of treatment delivery, number of sessions, theoretical basis) on intervention outcome will be explored in the pooled dataset. In addition, we will analyze intervention effects and moderators of effects in specific subgroups of interest (e.g., using only data from patients with low education, chronic medical conditions, etc.).

Method

General study approach

First, a systematic review will be performed to identify eligible papers, studies will be selected and corresponding authors will be contacted for each of the identified papers and asked to provide raw data from their study. The study will be completed in compliance with the PRISMA Statement. Individual patient data will be aggregated and a priori elected moderator variables will be analysed using a multilevel model approach.

Eligibility criteria

In this IPD MA, we will a) include randomized trials in which b) the effects of a psychological treatment were compared with a comparison group (waiting list, care-as-usual, psychological placebo, pill placebo, antidepressant medication) c) in adults d) with clinically relevant depressive symptoms e) but no major depressive disorder at baseline, f) assessed with a standardized diagnostic interview (see below) to exclude participants with full-blown mood disorder at baseline. Clinically relevant depressive symptoms will be defined as scoring above a cut-off score on a self-rating depression questionnaire; scoring above a cut-off score on a clinician-rated instrument; or meeting criteria for minor depression according to the Diagnostic and Statistical Manual of Mental Disorders

For peer review only

(DSM), International Classification of Diseases (ICD). We will also include studies in which participants with a diagnosed depressive disorder were examined and we will then exclude participants with a full-blown disorder on an individual basis using the primary data. No language restrictions will be applied.

Types of outcome measures

We will use the following types of outcome criteria: a) incidence of MDD, b) time to MDD onset, c) observer- and self-reported depression severity, d) response, e) remission, f) deterioration, g) quality of life, and h) anxiety. MDD will be measured by clinical interviews such as the SCID,[31] CIDI,[32] or MINI.[33] Depression severity will be measured using standardized depression outcome measures such as the Beck Depression Inventory (BDI),[34] Hamilton Depression Rating Scale (HAM-D),[35] or the Center for Epidemiological Depression Scale (CES-D).[36] If both observer-rated and self-report measures are used, we will explore intervention effects on both outcome measure types. If several observer-rated or self-report measures are used, preference will be given to the measures that are used the most across the different studies in order to increase comparability. If the type of outcome measures varies between studies, these measures will be transformed into standardized scores (using the common metric approach[37] or, if this is not possible, z-transformation). We will also dichotomize depression scores to explore effects on two response criteria (a 50% reduction in symptoms for relative change; a minimum absolute change in symptoms according to the reliable change index[38]) and remission (scoring below a predefined cut-off score). Deterioration rates will be calculated using a predefined absolute worsening of symptoms from baseline to follow up using the Reliable Change Index[40] and 50% symptom increase. Quality of life will be transformed to quality-adjusted life years (QALYs), using the British value set for EQ-5D utility values[39] and Brazier's algorithm for SF-6D utility values,[40] respectively. Anxiety severity will be measured using standardized self-report measures, such as the HADS[41] or BAI.[42] Note that we are planning to reduce the complexity for moderator analyses by only focusing on a) incidence of MDD and c) depression severity.

Moderators

We will investigate both moderators on individual patient-level (e.g., socio-demographic, clinical characteristics) as well as and on study level (e.g., type of treatment delivery, number of sessions, theoretical basis). Published papers are examined to identify potential moderators on patient level that have been assessed across studies. We will explore variables that have shown to predict

For peer review only

differential treatment outcome in psychological treatments for depression[43,44] and variables that are associated with depression onset.[45–47]

Clinical and personality characteristics that will be investigated, if sufficiently available, include depressive symptom severity,[48] lifetime-history of MDD,[49,50] number of previous depressive episodes,[49,51] anxiety symptoms,[49] comorbid mental health disorder (e.g. anxiety disorder)[50], previous exposure to depression treatment, family history of common mental health disorders,[50,52,53] global assessment of functioning, sleeping problems,[54–56] neuroticism,[48] recent life stress,[57] childhood adversities,[53] traumatic events,[58] significant life events (in the previous year),[59,60], daily hassles, emotion regulation,[61] poor self-perceived health (quality of life),[49,54,60] self-esteem,[62–64] (chronic) medical conditions,[55,56,65] physical functioning/ disability,[54] mastery, worrying, Body-Mass-Index, rumination, interpersonal problems,[51,60] body dissatisfaction,[64,66] physical activity level,[54,67] diet quality,[67] alcohol / substance use,[50,54,60] smoking,[54,65] resilience,[68] social support/ integration,[50,55,61,64] perceived social rejection/ mobbing. Sociodemographic variables that shall be examined are sex,[52,65,69,70] age,[50,69] education,[56,71] marital status,[71] relationship status,[69] living alone,[53] employment,[53] ethnicity (minority status),[72] economic deprivation / poverty,[55,60,72] parenthood (motherhood).[65] It is expected that not all studies that will be included assessed all variables. Hence, variables will only be examined if sufficient data across studies are available. Intervention characteristics that will be examined include the intervention format (individual, group, or guided self-help), the number of treatment sessions, overall treatment duration, session frequency,[73] the type of delivery (internet, face-to-face), the control condition (placebo/attention control, care as usual, waitlist, alternative treatment), type of psychotherapy (cognitive behaviour therapy, problem-solving, interpersonal or other type) and study quality.

Timing of outcome assessments

All post-intervention assessments will be pooled and treated as one assessment, despite varying time frames due to different intervention lengths. Treatment duration will be controlled for, if found to be associated with the dependent variable. We expect varying follow-up periods of the studies and will therefore categorize follow-ups into meaningful categories, such as follow-up that occurred 3-7 months (follow-up I), 8-13 months (follow up II), or over 14 months (follow-up III) after baseline.

Searches and Study Selection

For the identification of potential studies for inclusion, we will use a database of papers on the psychological treatment of depression described in detail elsewhere.[74] For this database, studies

For peer review only

have been identified from Pubmed, PsychInfo, Embase and the Cochrane Central Register of Controlled Trials. Additionally, previous meta-analyses of treatments for depression were screened for this database to ensure that no randomized trial was missed. These searches identified a total of 16,407 abstracts (12,196 after the removal of duplicates), from this 1,885 full text papers of RCTs on treatments for depression were retrieved for possible inclusion in the database. These papers will be then screened for inclusion in this meta-analysis. A further literature search will be conducted for studies published since the last update of the database. In addition, relevant authors in the field of depression prevention will be asked whether they are aware of any yet unpublished study that might fit the inclusion criteria.

Corresponding authors will be contacted for each of the identified papers and will be asked to provide raw data from their study. If an author does not respond after 1 month, a second attempt to contact them will be made. If the second contact fails, another author of the study will be contacted and invited to participate. A second attempt to contact this author will follow in another month if no response is received and so forth until a maximum of three authors were contacted. Study data will be considered unavailable in the event that no study authors have responded to multiple contact attempts or if all contacted authors indicate that they no longer have access to the data. If authors do not respond, are not able or not willing to share their data, we will compare these studies to the included ones in terms of design, participants, intervention, and quality.

Risk of bias assessment

The validity of the included studies will be assessed using four criteria from the Cochrane 'Risk of Bias' assessment tool.[75] This tool identifies possible sources of bias, including: the adequate generation of allocation sequence, the allocation concealment, blinding of assessors, and dealing with incomplete outcome data (this is assessed as positive when intention-to-treat analyses were conducted, meaning that all randomized patients were included in the analyses). Only data from published papers will be used to determine the risk of bias in order to use a consistent procedure across studies that does or does not share data. Two researchers will conduct the quality assessment independently and agreement rates will be reported. Disagreement will be solved through discussion.

Missing data

IPD MA will be conducted according to the intention-to-treat principle. Missing data is handled using a joint modeling approach to multiple imputation of individual participant data nested within studies.[76–78] In particular, we will use the R package jomo that uses Markov Chain Monte Carlo (MCMC) techniques to draw replacements for the missing values. [79] This procedure is based on a multilevel

For peer review only - http://bmjopen.bmj.com/site/about/guidelines.xhtml

imputation model that considers associations between continuous and categorical variables both at the level of participants (level 1) and studies (level 2). In addition, it allows for modeling between-study heterogeneity in the covariance matrices, which is especially useful when imputing variables that are completely missing from studies.[76] We will specify a multivariate empty imputation model including all available participant (level 1) and study (level 2) characteristics.[80] Assignment to intervention- vs. control-group will be used as a grouping variable in the imputation model to allow for treatment-specific intercept, variance and covariance parameters. Based on the final model we will generate at least 20 imputed data sets. The number of burn-in iterations and the number of iterations between imputed data sets will be chosen so that convergence can be ensured.[80] In the case of persistent convergence problems we will reduce the number of model parameters by dropping predictors and/or imposing constraints to the model (e.g., assuming a common level 1 covariance matrices across studies).

Analysis

<u>*Conventional meta-analysis on study level*</u>

We will first conduct a conventional meta-analysis, using data from the published papers. This will allow us to identify whether studies that did not provide data might bias the results of our IPD MA. This will be done by comparing those studies contributed to the IPD dataset to those who did not, with regard to the outcomes, risk of bias and score other study characteristics.

First, we will calculate the IRR for developing a depressive disorder in the intervention compared with the control group for each study based on published papers, and then pool the results using the Comprehensive Meta-Analysis Software package, version 3. With regard to effects on depression symptom severity, we will calculate Hedges' g as a measure of the effect size indicating the difference between the intervention and control conditions at post-treatment. These analyses will be done using a random-effects DerSimonian-Laird model[81] because considerable heterogeneity between studies is expected. To test homogeneity of effect sizes, we will calculate the I^2-statistic as an indicator of heterogeneity in percentages.[82] A value of 0% indicates no observed heterogeneity, and larger values indicate increasing heterogeneity, with 25% as low, 50% as moderate, and 75% as high. We will calculate 95% confidence intervals using the non-central chi-squared-based approach.[83] Small sample bias will be tested by inspecting the funnel plot visually, the Eggers test and we will apply Duval and Tweedie's trim-and-fill procedure[84] which yields an estimate of the effect size after small sample bias has been taken into account.[85]

For peer review only

IPD Meta-Analysis

For the IPD meta-analysis we will utilise a one-step data-analysis approach. This is currently the best possible meta-analysis approach with the standard two-step analysis being at best equivalent in some scenarios.[86] All models are repeated for all of the defined follow-ups.

Effects on MDD disorder onset: We will use multilevel logistic regression analysis based on the imputed datasets for predicting the occurrence of MDD from treatment group using R. Patient level data will be treated as level 1, study level data as level 2 in all further analyses. Models will include both random intercepts and random slopes to capture both unobserved heterogeneity in trial populations (intercept) and trial effectiveness (slope). We will proceed to calculate the odds ratio (OR) and its 95% intervals and also calculate the numbers needed to be treated (NNT) and its 95%-confidence intervals in order to avoid one additional MDD. In addition, we will conduct two additional analyses explicitly taking into account that observation periods and time to MDD onset may differ between participants or studies. To control for differences in observation periods, we will use multilevel binomial regression analysis with a complementary log-log link and offset for time since baseline, which provides an estimate of the treatment effect in terms of the IRR for developing a MDD [87]. To deal with differences in time to MDD onset we will use multilevel Cox proportional hazard models, which provide an estimate of the treatment effect in terms of the hazard ratio for developing a MDD.

Effects on symptom severity: We will use a multilevel regression analysis predicting standardised depression severity scores from treatment group and controlling for baseline depression severity. Again, we will include both a random intercept and random slope for the treatment effects to capture both unobserved heterogeneity between study populations (intercept) and study effectiveness (slope). Hedges' g will be calculated as an effect size measure. The same approach will be used for analyzing effects on other continous outcome measures including quality of life and anxiety.

Effects on response, remission and deterioration: The standard criteria for measuring response in psychotherapy outcome research for depression is a 50% reduction on a standardized depression measure.[88] However, it can be argued that in individuals with subclinical symptoms a relative reduction of 50% of symptoms might be clinically less meaningful compared to individuals with Major Depression. Hence we will additionally calculate response using a predefined absolute reduction in symptoms using the Reliable Change Index[38] Remission will be defined using standard cut-off scores of the respective instruments. Deterioration will be defined using a predefined absolute worsening of symptoms from baseline to follow up using the Reliable Change Index[38] and 50% symptom increase. Generally, event occurrence will be predicted from treatment group using

For peer review only

multilevel logistic regression analysis. We will proceed to calculate the OR and its 95% intervals and also calculate the numbers needed to be treated (NNT) and its 95%-confidence intervals in order to achieve one additional response, respectively remission as compared to the control group.[89]

Effects on deterioration rates:

Moderator Analyses: We will explore predictors of outcome (i.e., prognostic variables) and moderators of the intervention effect (i.e., prescriptive variables) by including selected participant-level and study level variables as well as their interaction with the intervention as additional predictors in the multilevel (logistic) regression analyses. These analyses will be based on the total sample (i.e., on the imputed datasets including all studies) and focus on predicting incidence of MDD, depression severity and symptom deterioration. Predictors will be selected based on the amount of available/missing data and the bivariate associations with outcome measures in the intervention- and control-group. In order to increase statistical power, moderator analyses on long-term effects will be done using combined follow up assessments in order that every included study contribute data to the analysis.

Subgroup Analyses: We also plan to examine the effectiveness of the interventions and moderators of treatment outcome in subgroups that are of special interest for tailoring prevention programs (e.g. older adults, low educated adults, minority status, mothers of new-borns, medical conditions, and individuals without lifetime history of depression). These analyses will be based on subsamples. Note that it will be necessary to generate new imputed datasets for these analyses to ensure congeniality with the imputation model.[78] The same strategy will be applied to investigate effects and moderators in specific intervention delivery forms (e.g., internet, guided/unguided self-help, group format). However, whether these and other analyses in subgroups of interest should be conducted depends on the number of studies/participants that are eligible.

Sensitivity Analyses: A number of sensitivity analyses will be conducted in order test the robustness of our findings. For example, we will run a separate model in which we exclude trials with high risk of bias. If a sufficient number of studies include the same outcome measurement (e.g., for depression severity), we will conduct separate analyses using only this specific outcome measurement, instead of using the standardized score. We will also run a complete-case analysis and compare the results to the intention-to-treat analysis in order to determine whether a difference exists between those that dropped out from the trials compared to those who persisted. Other sensitivity analyses may be necessary and will be decided on after all data have been collected and examined.

Discussion

For peer review only

The burden attributable to major depression is immense and although effective treatments are available, effects on disease burden is limited. Treatments so far failed to show that the prevalence of depression in the population can be reduced, even in those countries in which evidence-based treatments have been made widely available. Hence, new approaches are needed to reduce the burden of MDD at population level. This study will provide a precise estimate of the effects of indicated preventive interventions for subclinical symptoms of depression on short and long-term depression severity, MDD onset and other relevant outcome criteria. Using an individual patient-data meta-analytic approach we will be able to estimate specific effects in relevant subgroups of interest and test whether the effectiveness depends on individual participant criteria.

Such approaches have been used with some frequency in medicine, but are less often applied in the field of psychological treatment outcome research, although recently a number of studies have been published[90–96] and more seem to be on the way.[97–99] As the field moves towards personalized medicine, it is crucial to know specific effects for specific kinds of treatments for patients with certain characteristics, in order to select the best possible treatment for an individual patient. IPD MA allow this with sufficient statistical power.

However, such an approach has also a number of challenges. First, until such a study is published, it is very likely that the search is already outdated and more trials have already been published that could theoretically been included. This is due to the fact, that solely the processes of obtaining and integrating the primary data into one dataset, take very long. Updating the search and including additional datasets within the review process needs to be balanced to what can be gained by doing so with regard to the specific research question investigated, as theoretically this process could be done repeatedly. For example, if effects in relevant investigated subgroups are consistent across trials, heterogeneity is low, the number of included studies and participants is reasonable, effects are clinical meaningful with narrow confidence intervals for effect sizes, then it is unlikely that the inclusion of an additional study would result in meaningful changes that would justify the delay in publishing the results to be available for the scientific community and policy makers. On the other hand, if differences of effect sizes between specific subgroups are substantial, but moderator analyses are underpowered to detect such a difference and the inclusion of additional studies would change that, the potential additional value of updating the dataset potentially would outweigh the disadvantages. Second, a limitation of the IPD approach is that one very much relies with regard to investigating moderators of treatment outcome, on the variables that have been assessed in the primary study. In addition, many relevant predictors and moderators associated with depression onset or differential treatment response in the literature, such as for example lifetime history of depression, childhood adversities are not included in many of the published studies. However, recent advantages in statistics allow not only to account for between study heterogeneity when imputing

For peer review only - http://bmjopen.bmj.com/site/about/guidelines.xhtml

missing values but also to impute variables that are systematically missing in studies.[76,100] Nevertheless, we argue that authors should include variables in primary studies that potentially might explain heterogeneity of treatment effects, even when the study is not powered to reliable investigate differential treatment effects. This would allow using these data in IPD studies and might bring the field of precision medicine in psychological treatment outcome research substantially forward. Third, another challenge with IPD meta-analyses is that often not all available trials can be included in the dataset due to author non-response, lack of ethical approval to share the data or that data are not available anymore. This might introduce some bias, which is being addressed by comparing IPD findings with those of traditional meta-analyses in the present study.

For peer review only

Contributors

DDE, PC conceptualized and designed the study, PC contacted the primary authors, JR and CB are responsible for building the database. JZ is responsible for the data analyses. DDE drafted the manuscript; all authors critically revised the manuscript, read and approved the final version.

Funding

No specific funding for this work.

Competing interests

None declared.

Ethics

This paper is a study protocol for an individual patient data meta-analysis and does not require ethical approval. Anonymized data collected are managed by CB and JR and will be available for the complete research team.

Data sharing statement

Access to the data can be requested from the first author.

For peer review only

References

1 Alonso J, Angermeyer MC, Bernert S, *et al.* Prevalence of mental disorders in Europe: results from the European Study of the Epidemiology of Mental Disorders (ESEMeD) project. *Acta Psychiatr Scand Suppl* 2004;:21–7. doi:10.1111/j.1600-0047.2004.00327.x

2 Waraich P, Goldner EM, Somers JM, *et al.* Prevalence and incidence studies of mood disorders: a systematic review of the literature. *Can J Psychiatry* 2004;49:124–38.

3 Wittchen H-U, Jacobi F, Rehm J, *et al.* The size and burden of mental disorders and other disorders of the brain in Europe 2010. *Eur Neuropsychopharmacol* 2011;21:655–79. doi:10.1016/j.euroneuro.2011.07.018

4 Kessler RC, Chiu WT, Demler O, *et al.* Prevalence, severity, and comorbidity of 12-month DSM-IV disorders in the National Comorbidity Survey Replication. *Arch Gen Psychiatry* 2005;62:617–27. doi:10.1001/archpsyc.62.6.617

5 Saarni SI, Suvisaari J, Sintonen H, *et al.* Impact of psychiatric disorders on health-related quality of life: general population survey. *Br J Psychiatry* 2007;190:326–32. doi:10.1192/bjp.bp.106.025106

6 Ustün TB, Ayuso-Mateos JL, Chatterji S, *et al.* Global burden of depressive disorders in the year 2000. *Br J Psychiatry* 2004;184:386–92.

7 Berto P, D'Ilario D, Ruffo P, *et al.* Depression: cost-of-illness studies in the international literature, a review. *J Ment Health Policy Econ* 2000;3:3–10.

8 Greenberg PE, Birnbaum HG. The economic burden of depression in the US: societal and patient perspectives. *Expert Opin Pharmacother* 2005;6:369–76. doi:10.1517/14656566.6.3.369

9 Smit F, Cuijpers P, Oostenbrink J, *et al.* Costs of nine common mental disorders: implications for curative and preventive psychiatry. *J Ment Health Policy Econ* 2006;9:193–200.

10 Cuijpers P, van Straten A, Andersson G, *et al.* Psychotherapy for depression in adults: a meta-analysis of comparative outcome studies. *J Consult Clin Psychol* 2008;76:909–22. doi:10.1037/a0013075

11 Cuijpers P, Karyotaki E, Weitz E, *et al.* The effects of psychotherapies for major depression in adults on remission, recovery and improvement: A meta-analysis. *J Affect Disord* Published Online First: February 2014. doi:10.1016/j.jad.2014.02.026

12 Andrews G, Issakidis C, Sanderson K, *et al.* Utilising survey data to inform public policy:

For peer review only

comparison of the cost-effectiveness of treatment of ten mental disorders. *Br J Psychiatry* 2004;184:526–33.

13 Kohn R, Saxena S, Levav I, *et al.* The treatment gap in mental health care. *Bull World Health Organ* 2004;82:858–66. doi:/S0042-96862004001100011

14 Mack S, Jacobi F, Gerschler A, *et al.* Self-reported utilization of mental health services in the adult German population--evidence for unmet needs? Results of the DEGS1-Mental Health Module (DEGS1-MH). *Int J Methods Psychiatr Res* 2014;23:289–303. doi:10.1002/mpr.1438

15 Smith KLW, Matheson FI, Moineddin R, *et al.* Gender differences in mental health service utilization among respondents reporting depression in a national health survey. *Health (Irvine Calif)* 2013;5:1561–71. doi:10.4236/health.2013.510212

16 Cuijpers P, Beekman AT, Reynolds 3rd CF. Preventing depression: a global priority. *JAMA* 2012;307:1033–4. doi:10.1001/jama.2012.271

17 Munoz RF, Cuijpers P, Smit F, *et al.* Prevention of major depression. *Annu Rev Clin Psychol* 2010;6:181–212. doi:10.1146/annurev-clinpsy-033109-132040

18 van Zoonen K, Buntrock C, Ebert DD, *et al.* Preventing the onset of major depressive disorder: A meta-analytic review of psychological interventions. *Int J Epidemiol* 2014;43. doi:10.1093/ije/dyt175

19 Cuijpers P, de Graaf R, van Dorsselaer S. Minor depression: risk profiles, functional disability, health care use and risk of developing major depression. *J Affect Disord* 2004;79:71–9. doi:10.1016/s0165-0327(02)00348-8

20 Cuijpers P, Vogelzangs N, Twisk J, *et al.* Differential mortality rates in major and subthreshold depression: meta-analysis of studies that measured both. *Br J Psychiatry* 2013;202:22–7. doi:10.1192/bjp.bp.112.112169

21 Rucci P, Gherardi S, Tansella M, *et al.* Subthreshold psychiatric disorders in primary care: prevalence and associated characteristics. *J Affect Disord* 2003;76:171–81.

22 Goldney RD, Fisher LJ, Dal Grande E, *et al.* Subsyndromal depression: prevalence, use of health services and quality of life in an Australian population. *Soc Psychiatry Psychiatr Epidemiol* 2004;39:293–8. doi:10.1007/s00127-004-0745-5

23 Cuijpers P, Smit F, Oostenbrink J, *et al.* Economic costs of minor depression: a population-based study. *Acta Psychiatr Scand* 2007;115:229–36. doi:10.1111/j.1600-0447.2006.00851.x

24 Barbui C, Cipriani A, Patel V, *et al.* Efficacy of antidepressants and benzodiazepines in minor

For peer review only

depression: systematic review and meta-analysis. *Br J Psychiatry* 2011;198:11–6, sup 1. doi:10.1192/bjp.bp.109.076448

25 Cuijpers P, Koole SL, van Dijke A, *et al.* Psychotherapy for subclinical depression: meta-analysis. *Br J Psychiatry* 2014;205:268–74. doi:10.1192/bjp.bp.113.138784

26 Brookes ST, Whitely E, Egger M, *et al.* Subgroup analyses in randomized trials: risks of subgroup-specific analyses; power and sample size for the interaction test. *J Clin Epidemiol* 2004;57:229–36. doi:10.1016/j.jclinepi.2003.08.009

27 Clarke MJ. Individual patient data meta-analyses. *Best Pract Res Clin Obstet Gynaecol* 2005;19:47–55. doi:10.1016/j.bpobgyn.2004.10.011

28 Jones AP, Riley RD, Williamson PR, *et al.* Meta-analysis of individual patient data versus aggregate data from longitudinal clinical trials. *Clin Trials* 2009;6:16–27. doi:10.1177/1740774508100984

29 Riley RD, Lambert PC, Abo-Zaid G. Meta-analysis of individual participant data: rationale, conduct, and reporting. *BMJ* 2010;340:c221. doi:10.1136/bmj.c221

30 Cooper H, Patall EA. The relative benefits of meta-analysis conducted with individual participant data versus aggregated data. *Psychol Methods* 2009;14:165–76. doi:10.1037/a0015565

31 Lobbestael J, Leurgans M, Arntz A. Inter-rater reliability of the Structured Clinical Interview for DSM-IV Axis I Disorders (SCID I) and Axis II Disorders (SCID II). *Clin Psychol Psychother* 2011;18:75–9. doi:10.1002/cpp.693

32 Robins LN, Wing J, Wittchen HU, *et al.* The Composite International Diagnostic Interview. An epidemiologic Instrument suitable for use in conjunction with different diagnostic systems and in different cultures. *Arch Gen Psychiatry* 1988;45:1069–77.http://www.ncbi.nlm.nih.gov/pubmed/2848472 (accessed 9 Apr 2017).

33 Sheehan D V, Lecrubier Y, Sheehan KH, *et al.* The Mini-International Neuropsychiatric Interview (M.I.N.I.): the development and validation of a structured diagnostic psychiatric interview for DSM-IV and ICD-10. *J Clin Psychiatry* 1998;:22-33-57.http://www.ncbi.nlm.nih.gov/pubmed/9881538 (accessed 9 Apr 2017).

34 Beck AT, Steer A, Brown GK. *BDI-II: Beck Depression Inventory Manual. 2nd ed. San Antonio, TX: Psychological Corporation; 1996*. 2nd ed. San Antonio: : Psychological Corporation 1996.

35 HAMILTON M. A rating scale for depression. *J Neurol Neurosurg Psychiatry* 1960;23:56–62.

For peer review only

36 Radloff LS. The CES-D Scale: A Self-Report Depression Scale for Research in the General Population. *Appl Psychol Meas* 1977;1:385–401. doi:10.1177/014662167700100306

37 Wahl I, Löwe B, Bjorner JB, *et al.* Standardization of depression measurement: a common metric was developed for 11 self-report depression measures. *J Clin Epidemiol* 2014;67:73–86. doi:10.1016/j.jclinepi.2013.04.019

38 Jacobson NS, Truax P. Clinical significance: a statistical approach to defining meaningful change in psychotherapy research. *J Consult Clin Psychol* 1991;59:12–9.

39 Dolan P. Modeling valuations for EuroQol health states. *Med Care* 1997;35:1095–108.

40 Brazier JE, Rowen D, Hanmer J. Revised SF-6D scoring programmes: a summary of improvements. *PRO Newsl* 2008;40:14–5.

41 Bjelland I, Dahl AA, Haug TT, *et al.* The validity of the Hospital Anxiety and Depression Scale. An updated literature review. *J Psychosom Res* 2002;52:69–77.

42 de Lima Osório F, Crippa JAS, Loureiro SR. Further psychometric study of the Beck Anxiety Inventory including factorial analysis and social anxiety disorder screening. *Int J Psychiatry Clin Pract* 2011;15:255–62. doi:10.3109/13651501.2011.605955

43 Kessler RC, Van Loo HM, Wardenaar KJ, *et al.* Using patient self-reports to study heterogeneity of treatment effects in major depressive disorder. *Epidemiol Psychiatr Sci* 2017;26. doi:10.1017/S2045796016000020

44 Kessler RC, Van Loo HM, Wardenaar KJ, *et al.* Testing a machine-learning algorithm to predict the persistence and severity of major depressive disorder from baseline self-reports. *Mol Psychiatry* 2016;21. doi:10.1038/mp.2015.198

45 Nigatu YT, Liu Y, Wang J. External validation of the international risk prediction algorithm for major depressive episode in the US general population: the PredictD-US study. *BMC Psychiatry* 2016;16:256. doi:10.1186/s12888-016-0971-x

46 King M, Bottomley C, Bellón-Saameño JA, *et al.* An international risk prediction algorithm for the onset of generalized anxiety and panic syndromes in general practice attendees: predictA. *Psychol Med* 2011;41:1625–39. doi:10.1017/S0033291710002400

47 Liu Y, Sareen J, Bolton J, *et al.* Development and validation of a risk-prediction algorithm for the recurrence of panic disorder. *Depress Anxiety* 2015;32:341–8. doi:10.1002/da.22359

48 Støen Grotmol K, Gude T, Moum T, *et al.* Risk factors at medical school for later severe depression: A 15-year longitudinal, nationwide study (NORDOC). *J Affect Disord*

For peer review only

2013;146:106–11. doi:10.1016/j.jad.2012.08.047

49 Bromberger JT, Schott L, Kravitz HM, *et al.* Risk factors for major depression during midlife among a community sample of women with and without prior major depression: are they the same or different? *Psychol Med* 2015;45:1653–64. doi:10.1017/S0033291714002773

50 Hölzel L, Härter M, Reese C, *et al.* Risk factors for chronic depression — A systematic review. *J Affect Disord* 2011;129:1–13. doi:10.1016/j.jad.2010.03.025

51 Lewinsohn PM, Gotlieb IH, Seeley JR. Adolescent Psychopathology: IV. Specificity of Psychosocial Risk Factors for Depression and Substance Abuse in Older Adolescents. *J Am Acad Child Adolesc Psychiatry* 1995;34:1221–9. doi:10.1097/00004583-199509000-00021

52 Kounali D, Zammit S, Wiles N, *et al.* Common versus psychopathology-specific risk factors for psychotic experiences and depression during adolescence. *Psychol Med* 2014;44:2557–66. doi:10.1017/S0033291714000026

53 Heslin M, Desai R, Lappin JM, *et al.* Biological and psychosocial risk factors for psychotic major depression. *Soc Psychiatry Psychiatr Epidemiol* 2016;51:233–45. doi:10.1007/s00127-015-1131-1

54 Chang S-C, Pan A, Kawachi I, *et al.* Risk factors for late-life depression: A prospective cohort study among older women. *Prev Med (Baltim)* Published Online First: 2016. doi:10.1016/j.ypmed.2016.08.014

55 Chan MF, Zeng W. Exploring risk factors for depression among older men residing in Macau. *J Clin Nurs* 2011;20:2645–54. doi:10.1111/j.1365-2702.2010.03689.x

56 Zhou X, Bi B, Zheng L, *et al.* The prevalence and risk factors for depression symptoms in a rural Chinese sample population. *PLoS One* 2014;9:e99692. doi:10.1371/journal.pone.0099692

57 Whiteman K, Ruggiano N, Thomlison B. Transforming mental health services to address gender disparities in depression risk factors. *J Women Aging* 2016;:1–9. doi:10.1080/08952841.2015.1072027

58 Tang B, Liu X, Liu Y, *et al.* A meta-analysis of risk factors for depression in adults and children after natural disasters. *BMC Public Health* 2014;14:623. doi:10.1186/1471-2458-14-623

59 Nakulan A, Sumesh TP, Kumar S, *et al.* Prevalence and risk factors for depression among community resident older people in Kerala. *Indian J Psychiatry* 2015;57:262–6. doi:10.4103/0019-5545.166640

60 Salokangas RKR, Poutanen O. Risk factors for depression in primary care: findings of the

For peer review only

TADEP project. *J Affect Disord* 1998;48:171–80. doi:10.1016/S0165-0327(97)00171-7

61 Li J, Theng Y-L, Foo S. Depression and Psychosocial Risk Factors among Community-Dwelling Older Adults in Singapore. *J Cross Cult Gerontol* 2015;30:409–22. doi:10.1007/s10823-015-9272-y

62 Pelkonen M, Marttunen M, Kaprio J, *et al.* Adolescent risk factors for episodic and persistent depression in adulthood. A 16-year prospective follow-up study of adolescents. *J Affect Disord* 2008;106:123–31. doi:10.1016/j.jad.2007.06.001

63 MacPhee AR, Andrews JJW. Risk factors for depression in early adolescence. *Adolescence* 2006;41:435–66.

64 Czeglédi E, Urbán R. [Risk factors and alteration of depression among participants of an inpatient weight loss program]. *Psychiatr Hungarica A Magy Pszichiátriai Társaság tudományos folyóirata* 2012;27:361–78.

65 Yanzón de la Torre A, Oliva N, Echevarrieta PL, *et al.* Major depression in hospitalized Argentine general medical patients: Prevalence and risk factors. *J Affect Disord* 2016;197:36–42. doi:10.1016/j.jad.2016.02.066

66 Brausch AM, Gutierrez PM. The role of body image and disordered eating as risk factors for depression and suicidal ideation in adolescents. *Suicide Life Threat Behav* 2009;39:58–71. doi:10.1521/suli.2009.39.1.58

67 Hoare E, Skouteris H, Fuller-Tyszkiewicz M, *et al.* Associations between obesogenic risk factors and depression among adolescents: a systematic review. *Obes Rev* 2014;15:40–51. doi:10.1111/obr.12069

68 Wild J, Smith K V, Thompson E, *et al.* A prospective study of pre-trauma risk factors for post-traumatic stress disorder and depression. *Psychol Med* 2016;46:2571–82. doi:10.1017/S0033291716000532

69 Miletic V, Lukovic JA, Ratkovic N, *et al.* Demographic risk factors for suicide and depression among Serbian medical school students. *Soc Psychiatry Psychiatr Epidemiol* 2015;50:633–8. doi:10.1007/s00127-014-0950-9

70 Sajjadi H, Mohaqeqi Kamal SH, Rafiey H, *et al.* A systematic review of the prevalence and risk factors of depression among iranian adolescents. *Glob J Health Sci* 2013;5:16–27. doi:10.5539/gjhs.v5n3p16

71 Anstey KJ, von Sanden C, Sargent-Cox K, *et al.* Prevalence and risk factors for depression in a

For peer review only

longitudinal, population-based study including individuals in the community and residential care. *Am J Geriatr Psychiatry* 2007;15:497–505. doi:10.1097/JGP.0b013e31802e21d8

72 Lu W, Bian Q, Song Y, *et al.* Prevalence and related risk factors of anxiety and depression among Chinese college freshmen. *J Huazhong Univ Sci Technolog Med Sci* 2015;35:815–22. doi:10.1007/s11596-015-1512-4

73 Cuijpers P, Huibers M, Ebert DD, *et al.* How much psychotherapy is needed to treat depression? A metaregression analysis. *J Affect Disord* 2013;149:1–13. doi:10.1016/j.jad.2013.02.030

74 Cuijpers P, van Straten A, Warmerdam L, *et al.* Psychological treatment of depression: a meta-analytic database of randomized studies. *BMC Psychiatry* 2008;8:36. doi:10.1186/1471-244x-8-36

75 Higgins JM, Altman DG. Assessing Risk of Bias in included studies. In: *Cochrane Handbook for Systematic Reviews of Interventions:* John Wiley & Sons, Ltd. pp 2008. 187–241.

76 Quartagno M, Carpenter JR. Multiple imputation for IPD meta-analysis: allowing for heterogeneity and studies with missing covariates. *Stat Med* 2016;35:2938–54. doi:10.1002/sim.6837

77 Lüdtke O, Robitzsch A, Grund S. Multiple imputation of missing data in multilevel designs: A comparison of different strategies. *Psychol Methods* 2017;22:141–65. doi:10.1037/met0000096

78 Enders CK, Mistler SA, Keller BT. Multilevel multiple imputation: A review and evaluation of joint modeling and chained equations imputation. *Psychol Methods* 2016;21:222–40. doi:10.1037/met0000063

79 Quartagno M, Maintainer JC. R Package ‘jomo’ Multilevel Joint Modelling Multiple Imputation. Published Online First: 2016.https://cran.r-project.org/web/packages/jomo/jomo.pdf (accessed 9 Apr 2017).

80 Grund S, Lüdtke O, Robitzsch A. Multiple Imputation of Multilevel Missing Data. *SAGE Open* 2016;6:215824401666822. doi:10.1177/2158244016668220

81 DerSimonian R, Laird N. Meta-analysis in clinical trials. *Control Clin Trials* 1986;7:177–88.

82 Ioannidis JP, Patsopoulos NA, Evangelou E. Uncertainty in heterogeneity estimates in meta-analyses. *BMJ* 2007;335:914–6. doi:10.1136/bmj.39343.408449.80

83 Orsini N, Higgins J, Bottai M, *et al.* Heterogi: Stata module to quantify heterogeneity in a

For peer review only

Meta-analysis. 2013.

84 Duval S, Tweedie R. Trim and fill: A simple funnel-plot-based method of testing and adjusting for publication bias in meta-analysis. *Biometrics* 2000;56:455–63.

85 Borenstein M, Hedges L V., Higgins JPT, *et al. Introduction to Meta-Analysis*. Chichester, UK: : John Wiley & Sons, Ltd 2009. doi:10.1002/9780470743386

86 Mathew T, Nordström K. Comparison of one-step and two-step meta-analysis models using individual patient data. *Biom J* 2010;52:271–87. doi:10.1002/bimj.200900143

87 Martuzzi M, Elliott P. Estimating the incidence rate ratio in cross-sectional studies using a simple alternative to logistic regression. *Ann Epidemiol* 1998;8:52–5.http://www.ncbi.nlm.nih.gov/pubmed/9465994 (accessed 16 May 2017).

88 Rush AJ, Kraemer HC, Sackeim HA, *et al.* Report by the ACNP Task Force on response and remission in major depressive disorder. *Neuropsychopharmacology* 2006;31:1841–53. doi:10.1038/sj.npp.1301131

89 Laupacis A, Sackett DL, Roberts RS. An assessment of clinically useful measures of the consequences of treatment. *N Engl J Med* 1988;318:1728–33. doi:10.1056/NEJM198806303182605

90 Ebert DD, Donkin L, Andersson G, *et al.* Does Internet-based guided-self-help for depression cause harm? An individual participant data meta-analysis on deterioration rates and its moderators in randomized controlled trials. *Psychol Med* 2016;46. doi:10.1017/S0033291716001562

91 Bower P, Kontopantelis E, Sutton A, *et al.* Influence of initial severity of depression on effectiveness of low intensity interventions: meta-analysis of individual patient data. *BMJ* 2013;346:f540.

92 Karyotaki E, Riper H, Twisk J, *et al.* Efficacy of Self-guided Internet-Based Cognitive Behavioral Therapy in the Treatment of Depressive Symptoms. *JAMA Psychiatry* Published Online First: 22 February 2017. doi:10.1001/jamapsychiatry.2017.0044

93 Karyotaki E, Kleiboer A, Smit F, *et al.* Predictors of treatment dropout in self-guided web-based interventions for depression: an ‘individual patient data’ meta-analysis. *Psychol Med* 2015;45:2717–26. doi:10.1017/S0033291715000665

94 Cuijpers P, Weitz E, Twisk J, *et al.* Gender as predictor and moderator of outcome in cognitive behavior therapy and pharmacotherapy for adult depression: an "individual patient

For peer review only

data" meta-analysis. *Depress Anxiety* 2014;31:941–51. doi:10.1002/da.22328

95 Weitz ES, Hollon SD, Twisk J, *et al.* Baseline Depression Severity as Moderator of Depression Outcomes Between Cognitive Behavioral Therapy vs Pharmacotherapy: An Individual Patient Data Meta-analysis. *JAMA psychiatry* 2015;72:1102–9. doi:10.1001/jamapsychiatry.2015.1516

96 Bennett K, Manassis K, Walter SD, *et al.* COGNITIVE BEHAVIORAL THERAPY AGE EFFECTS IN CHILD AND ADOLESCENT ANXIETY: AN INDIVIDUAL PATIENT DATA METAANALYSIS. *Depress Anxiety* 2013;30:829–41. doi:10.1002/da.22099

97 Furukawa TA, Schramm E, Weitz ES, *et al.* Cognitive-Behavioural Analysis System of Psychotherapy (CBASP), a drug, or their combination: differential therapeutics for persistent depressive disorder: a study protocol of an individual participant data network meta-analysis. *BMJ Open* 2016;6:e011769. doi:10.1136/bmjopen-2016-011769

98 Purgato M, Gross AL, Jordans MJ, *et al.* Psychosocial interventions for children exposed to traumatic events in low- and middle-income countries: study protocol of an individual patient data meta-analysis. *Syst Rev* 2014;3:34. doi:10.1186/2046-4053-3-34

99 Weitz E, Kleiboer A, van Straten A, *et al.* Individual patient data meta-analysis of combined treatments versus psychotherapy (with or without pill placebo), pharmacotherapy or pill placebo for adult depression: a protocol. *BMJ Open* 2017;7:e013478. doi:10.1136/bmjopen-2016-013478

100 Jolani S, Debray TPA, Koffijberg H, *et al.* Imputation of systematically missing predictors in an individual participant data meta-analysis: a generalized approach using MICE. *Stat Med* 2015;34:1841–63. doi:10.1002/sim.6451

For peer review only

For peer review only - http://bmjopen.bmj.com/site/about/guidelines.xhtml

Efficacy and moderators of psychological interventions in treating subclinical symptoms of depression and preventing Major Depressive Disorder onsets: Protocol for an individual patient data meta-analysis of randomized controlled trials

Journal:	*BMJ Open*
Manuscript ID	bmjopen-2017-018582.R1
Article Type:	Protocol
Date Submitted by the Author:	12-Dec-2017
Complete List of Authors:	Ebert, David; Friedrich-Alexander University Erlangen Nuremberg, Clinical Psychology and Psychotherapy<br>Buntrock, Claudia; Friedrich-Alexander-Universitat Erlangen-Nurnberg, Clinical Psychology and Psychotherapy<br>Reins, Jo Annika; Leuphana Universitat Luneburg<br>Zimmermann, Johannes; Psychologische Hochschule Berlin<br>Cuijpers, Pim; VU University Amsterdam, Department of Clinical Psychology
**Primary Subject Heading**:	Mental health
Secondary Subject Heading:	Evidence based practice, Patient-centred medicine, Research methods, Health services research, Public health
Keywords:	MENTAL HEALTH, Depression & mood disorders < PSYCHIATRY, PREVENTIVE MEDICINE

Efficacy and moderators of psychological interventions in treating subclinical symptoms of depression and preventing Major Depressive Disorder onsets: Protocol for an individual patient data meta-analysis of randomized controlled trials

David Daniel Ebert[1]*, Claudia Buntrock[1], Jo Annika Reins[2], Johannes Zimmermann[3], Pim Cuijpers[4]

[1] Department of Clinical Psychology and Psychotherapy, Friedrich-Alexander-University Erlangen-Nuremberg, Naegelsbachstraße 25a, 91052 Erlangen, Germany

[2] Institute of Psychology, Leuphana University Lueneburg, Scharnhorststraße 1, 21335 Lueneburg, Germany

[3] Psychologische Hochschule Berlin, Am Köllnischen Park 2, 10179 Berlin, Germany

[4] Department of Clinical, Neuro and Developmental Psychology, EMGO+ Institute for Health and Care Research, VU University Amsterdam, Van der Boechorststraat 1, 1081 BT, Amsterdam, The Netherlands

*Corresponding author:

Dr. David Daniel Ebert

Friedrich-Alexander-University Erlangen-Nürnberg,

Department of Clinical Psychology and Psychotherapy

Naegelsbachstr. 25a, 91052 Erlangen, Germany

david.ebert@fau.de

Claudia Buntrock: claudia.buntrock@fau.de

Jo Annika Reins: reins@leuphana.de

Johannes Zimmermann: j.zimmermann@psychologische-hochschule.de

Pim Cuijpers: p.cuijpers@vu.nl

For peer review only - http://bmjopen.bmj.com/site/about/guidelines.xhtml

Abstract

Introduction

The long-term effectiveness of psychological interventions for the treatment of subthreshold depression and the prevention of depression is unclear and effects vary among subgroups of patients indicating that not all patients profit from such interventions. Randomized clinical trials are mostly underpowered to examine adequately subgroups and moderator effects. The aim of the present study is, therefore, to examine the short- and long-term as well as moderator effects of psychological interventions compared to control groups in adults with subthreshold depression on depressive symptom severity, treatment response, remission, symptom deterioration, quality of life, anxiety, and the prevention of major depressive disorder (MDD) onsets on individual patient- and study level using an individual-patient data meta-analysis approach.

Methods and analysis

Systematic searches in PubMed, PsycINFO, Embase and the Cochrane Central Register of Controlled Trials (CENTRAL) were conducted. We will use the following types of outcome criteria: a) onset of major depression, b) time to major depression onset, c) observer- and self-reported depressive symptom severity, d) response, e) remission, f) symptom deterioration, g) quality of life, h) anxiety, and i) suicidal thoughts and behaviors. Multilevel models with participants nested within studies will be used. Missing data will be handled using a joint modeling approach to multiple imputation. A number of sensitivity analyses will be conducted in order test the robustness of our findings.

Ethics and dissemination

The investigators of the primary trials have obtained ethical approval for the data used in the present study and for sharing the data, if this was necessary according to local requirements and was not covered from the initial ethic assessment.

This study will summarize the available evidence on the short- and long-term effectiveness of preventive psychological interventions for the treatment of subthreshold depression and prevention of major depressive disorder onset. Identification of subgroups of patients in which those interventions are most effective will guide the development of evidence-based personalized interventions for patients with subthreshold depression.

Systematic review registration

This study has been registered with the PROSPERO database (no. CRD42017058585).

For peer review only - http://bmjopen.bmj.com/site/about/guidelines.xhtml

For peer review only

For peer review only - http://bmjopen.bmj.com/site/about/guidelines.xhtml

Strengths and limitations of this study

- A strength of the presented individual patient-data meta-analysis (IPD MA) is that this approach allows sufficient statistical power to evaluate specific effects for specific kinds of treatments for patients with certain characteristics, in order to select the best possible treatment for an individual patient (i.e. personalized medicine).
- One limitation of the IPD MA is that while investigating moderators of treatment outcome, one very much relies on the variables that have been assessed in the primary studies. However, many of the relevant predictors and moderators associated with depression onset or differential treatment response reported in the literature were not assessed in the included studies.
- Another limitation of the IPD MA approach is that some bias is introduced because not all eligible trials can be included in the analyses due to author non-response, lack of ethical approval to share the data or that data are not available anymore.

For peer review only

For peer review only - http://bmjopen.bmj.com/site/about/guidelines.xhtml

Introduction

Major Depressive Disorder (MDD) is highly prevalent,[1–4] associated with substantial impairment[5,6] and economic costs.[7–9] Psychological treatments have been shown to be effective in the treatment of depression.[10,11] However, it has been estimated that even under the hypothetical scenario of full coverage with and adherence to evidence-based treatments approximately only one third of the disease burden attributable to MDD could be averted.[12] Moreover, in practice the majority of depressed people remain untreated[3,13], even in high income countries.[14,15]

Therefore, attention has increasingly been shifted to the prevention of MDD onsets.[16,17] One specific form of prevention is indicated prevention. In such interventions subthreshold symptoms are treated in order to prevent the transition to a full-blown depressive disorder.[17] Meta-analytic evidence shows that indicated psychological preventive approaches can be effective in preventing depressive episodes.[18] The latest systematic review, which included randomized trials that have been published up to March 2012, found psychological interventions for subclinical symptoms to be effective in reducing the risk of developing a MDD at 6-month (Incidence rate ratio[IRR] = 0.61; 5 studies)) and 12-month follow-up (IRR = 0.74; 4 studies). Since then, many more randomized controlled trials have been published, warranting an update of the evidence.

Moreover, the treatment of subclinical symptoms of depression itself is relevant. Subthreshold depressive symptoms are highly prevalent,[19] related to increased mortality,[20] poorer quality of life,[21] increased health care service utilisation,[22] and vast economic costs.[23] However, results for the treatment of subclinical symptoms are yet conflicting. Pharmacological interventions are unlikely to have a clinical advantage over placebos in treating subthreshold depression.[24] In addition, although a recent meta-analysis found small-to-moderate effect sizes for psychological interventions on depressive symptom severity at post-treatment compared to usual care,[25] four studies using clinician-rated outcomes did not indicate significant positive results. [26] Moreover, we are not aware of any systematic review exploring the long-term effects of treatments for subclinical symptoms with regard to depressive symptom severity, and effects on other relevant outcomes such as anxiety or quality of life have not been examined.

Another issue not yet addressed is the possibility that the effectiveness of psychological interventions for subthreshold depression varies across patients and not all subgroups of patients profit from such interventions. Given that the number of people from specific subgroups is often small in single trials, and randomized trials are usually powered to detect overall treatment effects, RCTs are mostly underpowered to perform adequately subgroup and moderator analyses.[27] As

For peer review only - http://bmjopen.bmj.com/site/about/guidelines.xhtml

studies also seldom report effectiveness for different patient characteristics, it is impossible to examine patient-level moderators using traditional meta-analytic approaches.

Individual participant data meta-analyses (IPD MA) can overcome some of the limitations of the conventional MAs on study level.[28–30] By pooling the primary data of individual trials, it is possible to conduct analyses not reported in original studies and obtain large enough sample sizes with sufficient power to examine effects in relevant subgroups and identify outcome moderators.[31]

The present study aims to examine the short- and long-term effects of psychological interventions compared to control groups in adults with subthreshold depression on depressive symptom severity, treatment response, remission, symptom deterioration, quality of life, anxiety, and the prevention of MDD onsets using an IPD MA approach. Moderators on individual patient-level (e.g., socio-demographic, clinical characteristics) and study level (e.g., type of treatment delivery, number of sessions, theoretical basis) on intervention outcome will be explored in the pooled dataset. In addition, we will analyze intervention effects and moderators of effects in specific subgroups of interest (e.g., using only data from patients with low education, chronic medical conditions, etc.).

Method

General study approach

First, a systematic review is performed to identify eligible papers. Corresponding authors of selected studies will be contacted and asked to provide raw data from their studies. The current study will be completed in compliance with the PRISMA Statement. Individual patient data will be aggregated and a priori defined moderator variables will be analysed using a multilevel model approach.

Eligibility criteria

In this IPD MA, we will a) include randomized trials in which b) the effects of a psychological treatment (delivered individually, in a group-, bibliotherapy, internet-based format) were compared with a comparison group (waiting list, care-as-usual, psychological placebo, pill placebo, antidepressant medication) c) in adults d) with clinically relevant depressive symptoms e) but no major depressive disorder at baseline, f) assessed with a standardized diagnostic interview (see below) to exclude participants with full-blown mood disorder at baseline. Psychological interventions are defined as the application of psychological mechanisms and interpersonal stances derived from psychological principles for the purpose of assisting people to modify their behaviours, cognitions, emotions, and/or other personal characteristics in directions that the participants deem desirable

For peer review only - http://bmjopen.bmj.com/site/about/guidelines.xhtml

[32,33].Clinically relevant depressive symptoms will be defined as scoring above a cut-off score on a self-rating depression questionnaire; scoring above a cut-off score on a clinician-rated instrument; or meeting criteria for minor depression according to the Diagnostic and Statistical Manual of Mental Disorders (DSM),or the International Classification of Diseases (ICD). We will also include studies in which participants with a diagnosed depressive disorder were examined and we will then exclude participants with a full-blown disorder on an individual basis using the primary data. No language restrictions will be applied.

Types of outcome measures

We will use the following types of outcome criteria: a) onset of MDD, b) time to MDD onset, c) observer- and self-reported depressive symptom severity, d) response, e) remission, f) symptom deterioration, g) quality of life, h) anxiety, and i) suicidal thoughts and behavior. MDD will be assessed with clinical interviews such as the SCID,[34] CIDI,[35] or MINI.[36] Depressive symptom severity will be measured using standardized depression outcome measures such as the Beck Depression Inventory (BDI),[37] Hamilton Depression Rating Scale (HAM-D),[38] or the Center for Epidemiological Depression Scale (CES-D).[39] If both observer-rated and self-report measures are available, we will explore intervention effects on both outcome measure types. If several observer-rated or self-report measures are used, preference will be given to the mostly used measures across the different studies in order to increase comparability. If the type of outcome measures varies between studies, these measures will be transformed into standardized scores (using the common metric approach[40] or, if this is not possible, z-transformation). We will also dichotomize scores on depressive symptoms to explore effects on two response criteria (a 50% reduction in symptoms for relative change; a minimum absolute change in symptoms according to the reliable change index[41]) and remission (scoring below a predefined cut-off score). Symptom deterioration rates will be calculated using a predefined absolute worsening of symptoms from baseline to follow-up using the Reliable Change Index[40] and 50% symptom increase. Quality of life will be transformed to quality-adjusted life years (QALYs), using, if possible, the British value set for EQ-5D-3L utility values[42] and Brazier's algorithm for SF-6D utility values,[43] respectively. Anxiety severity will be measured using standardized self-report measures, such as the HADS[44] or BAI.[45] Note that we are planning to reduce the complexity for moderator analyses by only focusing on a) onset of MDD and c) depressive symptom severity.

Moderators

For peer review only - http://bmjopen.bmj.com/site/about/guidelines.xhtml

We will investigate both moderators on individual patient-level (e.g., socio-demographic, clinical characteristics) as well as and on study level (e.g., type of treatment delivery, number of sessions, theoretical basis). Published papers are examined to identify potential moderators on patient level that have been assessed across studies. We will explore variables that have shown to predict differential treatment outcome in psychological treatments for depression[46,47] and variables that are associated with depression onset.[48–50]

Clinical and personality characteristics that shall be investigated include depressive symptom severity,[51] lifetime-history of MDD,[52,53] number of previous depressive episodes,[52,54] anxiety symptoms,[52] comorbid mental health disorder (e.g. anxiety disorder)[53], previous exposure to depression treatment, family history of common mental health disorders,[53,55,56] global assessment of functioning, sleeping problems,[57–59] neuroticism,[51] recent life stress,[60] childhood adversities,[56] traumatic events,[61] significant life events (in the previous year),[62,63], daily hassles, emotion regulation,[64] poor self-perceived health (quality of life),[52,57,63] self-esteem,[65–67] (chronic) medical conditions,[58,59,68] physical functioning/ disability,[57] mastery, worrying, Body-Mass-Index, rumination, interpersonal problems,[54,63] body dissatisfaction,[67,69] physical activity level,[57,70] diet quality,[70] alcohol / substance use,[53,57,63] smoking,[57,68] resilience,[71] social support/ integration,[53,58,64,67] perceived social rejection/ mobbing. Sociodemographic variables that shall be examined are sex,[55,68,72,73] age,[53,72] education,[59,74] marital status,[74] relationship status,[72] living alone,[56] employment,[56] ethnicity (minority status),[75] economic deprivation / poverty,[58,63,75] parenthood (motherhood).[68] It is expected that not all studies that will be included assessed all variables. Hence, a precondition for including a variable as a moderator in the actual analyses is availability of sufficient data . Intervention characteristics that will be examined include the intervention format (individual, group, or guided self-help), the number of treatment sessions, overall treatment duration, session frequency,[76] the type of delivery (internet, face-to-face), the control condition (placebo/attention control, care as usual, waitlist, alternative treatment), type of psychotherapy (cognitive behaviour therapy, problem-solving, interpersonal or other type) and study quality.

Timing of outcome assessments

All post-intervention assessments will be pooled and treated as one assessment, despite varying time frames in included studies. Treatment duration will be controlled for, if found to be associated with the dependent variable. We expect varying follow-up periods of the studies and will therefore categorize follow-ups into meaningful categories, such as follow-up that occurred 3-7 months (follow-up I), 8-13 months (follow up II), or over 14 months (follow-up III) after baseline.

For peer review only - http://bmjopen.bmj.com/site/about/guidelines.xhtml

Searches and Study Selection

For the identification of potential studies for inclusion, we will use a database of papers on the psychological treatment of depression described in detail elsewhere.[77] For this database, studies have been identified from Pubmed, PsychInfo, Embase and the Cochrane Central Register of Controlled Trials. In addition, previous meta-analyses of treatments for depression were screened for this database to ensure that no randomized trial was missed. These searches identified a total of 16,407 abstracts (12,196 after the removal of duplicates), from this 1,885 full text papers of RCTs on treatments for depression were retrieved for possible inclusion in the database. These papers will be screened for inclusion in this meta-analysis. A further literature search will be conducted for studies published since the last update of the database (studies published up to December 2017 will be considered for inclusion). In addition, relevant authors in the field of depression prevention will be asked whether they are aware of any yet unpublished study that might fit the inclusion criteria.

Corresponding authors will be contacted for each of the identified papers and will be asked to provide raw data from their study. If an author does not respond after 1 month, a second attempt to contact him/her will be made. If the second contact fails, another author of the study will be contacted and invited to participate. A second attempt to contact this author will follow a month later if no response is received and so forth until a maximum of three authors were contacted. Study data will be considered unavailable in the event that no study author has responded to multiple contact attempts or if all contacted authors indicate that they no longer have access to the data. If authors do not respond, are not able or not willing to share their data, we will compare these studies to the included ones in terms of design, participants, intervention, and quality.

Risk of bias assessment

The validity of the included studies will be assessed using four criteria from the Cochrane 'Risk of Bias' assessment tool.[78] This tool identifies possible sources of bias, including: the adequate generation of allocation sequence, the allocation concealment, blinding of assessors, and dealing with incomplete outcome data (this is assessed as positive when intention-to-treat analyses were conducted, meaning that all randomized participants were included in the analyses). Only data from published papers will be used to determine the risk of bias in order to use a consistent procedure across studies that does or does not share data. Two researchers will conduct the quality assessment independently and agreement rates will be reported. Disagreement will be solved through discussion.

For peer review only - http://bmjopen.bmj.com/site/about/guidelines.xhtml

Missing data

IPD MA will be conducted according to the intention-to-treat principle. Missing data is handled using a joint modeling approach to multiple imputation of individual participant data nested within studies.[79–81] In particular, we will use the R package jomo that uses Markov Chain Monte Carlo (MCMC) techniques to draw replacements for the missing values. [82] This procedure is based on a multilevel imputation model that considers associations between continuous and categorical variables both at the level of participants (level 1) and studies (level 2). In addition, it allows for modeling between-study heterogeneity in the covariance matrices, which is especially useful when imputing variables that are completely missing from studies.[79] We will specify a multivariate empty imputation model including all available participant (level 1) and study (level 2) characteristics.[83] Assignment to intervention- vs. control-group will be used as a grouping variable in the imputation model to allow for treatment-specific intercept, variance and covariance parameters. Based on the final model, we will generate at least 20 imputed data sets. The number of burn-in iterations and the number of iterations between imputed data sets will be chosen so that convergence can be ensured.[83] In the case of persistent convergence problems, we will reduce the number of model parameters by dropping predictors and/or imposing constraints to the model (e.g., assuming a common level 1 covariance matrices across studies).

Analysis

<u>*Conventional meta-analysis on study level*</u>

We will first conduct a conventional meta-analysis, using data from the published papers. This will enable us to examine whether studies that did not provide data might bias the results of our IPD MA. This will be done by comparing those studies contributed to the IPD dataset to those who did not with regard to the outcomes, risk of bias and other study characteristics.

First, we will calculate the IRR for developing a depressive disorder in the intervention compared to the control group for each study based on published papers, and then pool the results using the Comprehensive Meta-Analysis Software package, version 3. With regard to effects on depressive symptom severity, we will calculate Hedges' g as a measure of the effect size indicating the difference between the intervention and control conditions at post-treatment. These analyses will be done using a random-effects DerSimonian-Laird model[84] because considerable heterogeneity between studies is expected. To test homogeneity of effect sizes, we will calculate the I^2-statistic as an indicator of heterogeneity in percentages.[83] A value of 0-40% indicates unimportant heterogeneity, and larger values indicate increasing heterogeneity, with 30-60% as moderate, 50-90% substantial and 75-100% as considerable. We will calculate 95% confidence intervals using the

For peer review only - http://bmjopen.bmj.com/site/about/guidelines.xhtml

non-central chi-squared-based approach.[85] Small sample bias will be tested by inspecting the funnel plot visually, the Eggers test, and Duval and Tweedie's trim-and-fill procedure,[86] which yields an estimate of the effect size after small sample bias has been taken into account.[87]

IPD Meta-Analysis

For the IPD meta-analysis we will utilise a one-step data-analysis approach. This is currently the best possible meta-analysis approach with the standard two-step analysis being at best equivalent in some scenarios.[88] All models are repeated for all of the defined follow-ups.

Effects on MDD onset: We will use multilevel logistic regression analysis based on the imputed datasets for predicting the occurrence of MDD, including the assignment to intervention- vs. control-group as the focal predictor. atient level data will be treated as level 1 and study level data as level 2. Models will include both random intercepts and random slopes to capture both unobserved heterogeneity in trial populations (intercept) and trial effectiveness (slope). We will calculate odds ratios (OR) and corresponding 95% confidence intervals and also calculate the numbers needed to treat (NNT) and corresponding 95% confidence intervals in order to avoid one additional MDD. In addition, we will conduct two additional analyses taking varying observation periods and time to MDD onset explicitly into account. To control for differences in observation periods, we will use multilevel binomial regression analysis with a complementary log-log link and offset for time since baseline, which provides an estimate of the treatment effect in terms of the IRR for developing a MDD.[89] To assess differences in time to MDD onset, we will use multilevel Cox proportional hazard models, which provide an estimate of the treatment effect in terms of the hazard ratio for developing a MDD.

Effects on symptom severity: We will predict standardised depressive symptom severity scores from intervention- vs. control- group and control for baseline depressive symptom severity using a multilevel linear regression analysis. Again, we will include both a random intercept and random slope for treatment effects to capture both unobserved heterogeneity between study populations (intercept) and study effectiveness (slope). Hedges' g will be calculated as an effect size measure. The same approach will be used for analyzing effects on other continous outcome measures including quality of life and anxiety and suicidal ideation.

Effects on response, remission and symptom deterioration: The standard criterion for measuring response in psychotherapy outcome research for depression is a 50% reduction on a standardized depression measure.[90] However, it can be argued that in individuals with subclinical symptoms a relative reduction of 50% of symptoms might be clinically less meaningful compared to individuals

For peer review only - http://bmjopen.bmj.com/site/about/guidelines.xhtml

with major depression. Hence, we will additionally calculate response using a predefined absolute reduction in symptoms using the Reliable Change Index[41] Remission will be defined using standard cut-off scores of the respective instruments. Symptom deterioration will be defined using a predefined absolute worsening of symptoms from baseline to follow up using the Reliable Change Index[41] and 50% symptom increase. Generally, event occurrence will be predicted from treatment group using multilevel logistic regression analysis. We will proceed to calculate the OR and its 95% confidence intervals and also calculate the numbers needed to treat (NNT) and its 95%confidence intervals in order to achieve one additional response, respectively remission as compared to the control group.[91]

Moderator Analyses: We will explore predictors of outcome (i.e., prognostic variables) and moderators of the intervention effect (i.e., prescriptive variables) by including selected participant-level and study level variables as well as their interactions with the intervention as additional predictors in the multilevel (logistic) regression analyses. These analyses will be based on the total sample (i.e., on the imputed datasets including all studies) and focus on predicting onset of MDD, depressive symptom severity and symptom deterioration. Variables will be selected based on the combination of multiple criteria, including the amount of available/missing data, the bivariate associations with outcome measures in the intervention- and control-group, and the convergence of the multiple imputation model. In order to increase statistical power, moderator analyses on long-term effects will be done using combined follow-up assessments to include all studies that contribute follow-up data.

Subgroup Analyses: We also plan to examine the effectiveness of the interventions and moderators of treatment outcome in subgroups that are of special interest for tailoring prevention programs (e.g. older adults, low educated adults, minority status, mothers of new-borns, medical conditions, and individuals without lifetime history of depression). These analyses will be based on subsamples. Note that it will be necessary to generate new imputed datasets for these analyses to ensure congeniality with the imputation model.[81] The same strategy will be applied to investigate effects and moderators in specific intervention delivery forms (e.g., internet, guided/unguided self-help, group format). However, whether these and other analyses in subgroups of interest should be conducted depends on the number of studies/participants that are eligible.

Sensitivity Analyses: A number of sensitivity analyses will be conducted in order to test the robustness of our findings. For example, we will run a separate model in which we exclude trials with high risk of bias. If a sufficient number of studies include the same outcome measurement (e.g., for depressive symptom severity), we will conduct separate analyses using only this specific outcome measurement, instead of using the standardized score. We will also run a complete-case analysis and

For peer review only

For peer review only - http://bmjopen.bmj.com/site/about/guidelines.xhtml

compare the results to the intention-to-treat analysis in order to determine whether a difference exists between those that dropped out from the trials compared to those who persisted. Other sensitivity analyses may be necessary and will be decided on after all data have been collected and examined.

Discussion

The burden attributable to major depression is immense and although effective treatments are available, effects on disease burden is limited. Treatments so far failed to show that the prevalence of depression in the population can be reduced, even in those countries in which evidence-based treatments have been made widely available. Hence, new approaches are needed to reduce the burden of MDD at population level. This study will provide a precise estimate of the effects of indicated preventive interventions for subclinical symptoms of depression on short- and long-term depressive symptom severity, MDD onset and other relevant outcome criteria. Using an individual patient-data meta-analytic approach, we will be able to estimate specific effects in relevant subgroups of interest and test whether the effectiveness depends on individual participant criteria.

Such approaches have been used with some frequency in medicine, but are less often applied in the field of psychological treatment outcome research, although recently a number of studies have been published[92–98] or are in preparation.[99–101] As the field moves towards personalized medicine, it is crucial to know specific effects for specific kinds of treatments for patients with certain characteristics in order to select the best possible treatment for an individual patient. IPD MA allows to do this with sufficient statistical power.

However, such an approach also has a number of challenges. First, until such a study is published, it is very likely that the search is already outdated and more trials have already been published that could theoretically been included. This is due to the fact that solely the process of obtaining and integrating primary data into one dataset takes very long. Updating the search and including additional datasets within the review process needs to be balanced to what can be gained by doing so with regard to the specific research question investigated, as theoretically this process could be done repeatedly. For example, if effects in relevant investigated subgroups are consistent across trials, heterogeneity is low, the number of included studies and participants is reasonable, effects are clinical meaningful with narrow confidence intervals for effect sizes, then it is unlikely that the inclusion of an additional study would result in meaningful changes that would justify the delay in publishing the results to be available for the scientific community and policy makers. On the other hand, if differences of effect sizes between specific subgroups are substantial, but moderator analyses are underpowered to detect such a difference and the inclusion of additional studies would change this, the additional

For peer review only - http://bmjopen.bmj.com/site/about/guidelines.xhtml

value of updating the dataset would potentially outweigh the disadvantages. Second, a limitation of the IPD MA approach is that one very much relies on the variables that have been assessed in the primary study. In addition, many relevant predictors and moderators associated with depression onset or differential treatment response in the literature, such as for example lifetime history of depression, childhood adversities are not included in many of the published studies. However, recent advantages in statistics allow not only to account for between study heterogeneity when imputing missing values but also to impute variables that are systematically missing in studies.[79,102] Nevertheless, we argue that authors should include variables in primary studies that might eventually explain heterogeneity of treatment effects, even when the study is not powered to reliable investigate differential treatment effects. This would allow using these data in IPD MA studies and might bring the field of precision medicine in psychological treatment outcome research substantially forward. Third, another challenge with IPD MA is that often not all available trials can be included in the dataset due to author non-response, lack of ethical approval to share the data or that data are not available anymore. This might introduce some bias, which is being addressed by comparing IPD findings with those of traditional meta-analyses in the present study.

Ethics and dissemination

This paper is a study protocol for an individual patient data meta-analysis and does not require ethical approval. The investigators of the primary trials have obtained ethical approval for the data used in the present study and for sharing the data, if this was necessary according to local requirements and was not covered from the initial ethic assessment. Only anonymized data are included in the dataset which does not allow the identification of individual trial participants. Anonymized data collected are managed by CB and JR and will be available for the complete research team. External research can request access to the dataset for secondary analyses after publication of the results specified in this protocol, if local requirement of the original data should allow this.

This study will summarize the available evidence on the short- and long-term effectiveness of preventive psychological interventions for the treatment of subthreshold depression and prevention of major depressive disorder onset. Identification of subgroups of patients in which those interventions are most effective will guide the development of evidence-based personalized interventions for patients with subthreshold depression.

Authors' contributors

For peer review only - http://bmjopen.bmj.com/site/about/guidelines.xhtml

DDE, PC conceptualized and designed the study, PC contacted the primary authors, JR and CB are responsible for building the database. JZ is responsible for the data analyses. DDE drafted the manuscript and is guarantor of the review; all authors critically revised the manuscript, read and approved the final version.

Funding

No specific funding for this work.

Competing interests

None declared.

Data sharing statement

Access to the data can be requested from the first author.

For peer review only

References

1 Alonso J, Angermeyer MC, Bernert S, *et al.* Prevalence of mental disorders in Europe: results from the European Study of the Epidemiology of Mental Disorders (ESEMeD) project. *Acta Psychiatr Scand Suppl* 2004;:21–7. doi:10.1111/j.1600-0047.2004.00327.x

2 Waraich P, Goldner EM, Somers JM, *et al.* Prevalence and incidence studies of mood disorders: a systematic review of the literature. *Can J Psychiatry* 2004;49:124–38.

3 Wittchen H-U, Jacobi F, Rehm J, *et al.* The size and burden of mental disorders and other disorders of the brain in Europe 2010. *Eur Neuropsychopharmacol* 2011;21:655–79. doi:10.1016/j.euroneuro.2011.07.018

4 Kessler RC, Chiu WT, Demler O, *et al.* Prevalence, severity, and comorbidity of 12-month DSM-IV disorders in the National Comorbidity Survey Replication. *Arch Gen Psychiatry* 2005;62:617–27. doi:10.1001/archpsyc.62.6.617

5 Saarni SI, Suvisaari J, Sintonen H, *et al.* Impact of psychiatric disorders on health-related quality of life: general population survey. *Br J Psychiatry* 2007;190:326–32. doi:10.1192/bjp.bp.106.025106

6 Ustün TB, Ayuso-Mateos JL, Chatterji S, *et al.* Global burden of depressive disorders in the year 2000. *Br J Psychiatry* 2004;184:386–92.

7 Berto P, D'Ilario D, Ruffo P, *et al.* Depression: cost-of-illness studies in the international literature, a review. *J Ment Health Policy Econ* 2000;3:3–10.

8 Greenberg PE, Birnbaum HG. The economic burden of depression in the US: societal and patient perspectives. *Expert Opin Pharmacother* 2005;6:369–76. doi:10.1517/14656566.6.3.369

9 Smit F, Cuijpers P, Oostenbrink J, *et al.* Costs of nine common mental disorders: implications for curative and preventive psychiatry. *J Ment Health Policy Econ* 2006;9:193–200.

10 Cuijpers P, van Straten A, Andersson G, *et al.* Psychotherapy for depression in adults: a meta-analysis of comparative outcome studies. *J Consult Clin Psychol* 2008;76:909–22. doi:10.1037/a0013075

11 Cuijpers P, Karyotaki E, Weitz E, *et al.* The effects of psychotherapies for major depression in adults on remission, recovery and improvement: A meta-analysis. *J Affect Disord* Published Online First: February 2014. doi:10.1016/j.jad.2014.02.026

For peer review only - http://bmjopen.bmj.com/site/about/guidelines.xhtml

12 Andrews G, Issakidis C, Sanderson K, *et al.* Utilising survey data to inform public policy: comparison of the cost-effectiveness of treatment of ten mental disorders. *Br J Psychiatry* 2004;184:526–33.

13 Kohn R, Saxena S, Levav I, *et al.* The treatment gap in mental health care. *Bull World Health Organ* 2004;82:858–66. doi:/S0042-96862004001100011

14 Mack S, Jacobi F, Gerschler A, *et al.* Self-reported utilization of mental health services in the adult German population--evidence for unmet needs? Results of the DEGS1-Mental Health Module (DEGS1-MH). *Int J Methods Psychiatr Res* 2014;23:289–303. doi:10.1002/mpr.1438

15 Smith KLW, Matheson FI, Moineddin R, *et al.* Gender differences in mental health service utilization among respondents reporting depression in a national health survey. *Health (Irvine Calif)* 2013;5:1561–71. doi:10.4236/health.2013.510212

16 Cuijpers P, Beekman AT, Reynolds 3rd CF. Preventing depression: a global priority. *JAMA* 2012;307:1033–4. doi:10.1001/jama.2012.271

17 Munoz RF, Cuijpers P, Smit F, *et al.* Prevention of major depression. *Annu Rev Clin Psychol* 2010;6:181–212. doi:10.1146/annurev-clinpsy-033109-132040

18 van Zoonen K, Buntrock C, Ebert DD, *et al.* Preventing the onset of major depressive disorder: A meta-analytic review of psychological interventions. *Int J Epidemiol* 2014;43. doi:10.1093/ije/dyt175

19 Cuijpers P, de Graaf R, van Dorsselaer S. Minor depression: risk profiles, functional disability, health care use and risk of developing major depression. *J Affect Disord* 2004;79:71–9. doi:10.1016/s0165-0327(02)00348-8

20 Cuijpers P, Vogelzangs N, Twisk J, *et al.* Differential mortality rates in major and subthreshold depression: meta-analysis of studies that measured both. *Br J Psychiatry* 2013;202:22–7. doi:10.1192/bjp.bp.112.112169

21 Rucci P, Gherardi S, Tansella M, *et al.* Subthreshold psychiatric disorders in primary care: prevalence and associated characteristics. *J Affect Disord* 2003;76:171–81.

22 Goldney RD, Fisher LJ, Dal Grande E, *et al.* Subsyndromal depression: prevalence, use of health services and quality of life in an Australian population. *Soc Psychiatry Psychiatr Epidemiol* 2004;39:293–8. doi:10.1007/s00127-004-0745-5

23 Cuijpers P, Smit F, Oostenbrink J, *et al.* Economic costs of minor depression: a population-based study. *Acta Psychiatr Scand* 2007;115:229–36. doi:10.1111/j.1600-0447.2006.00851.x

For peer review only - http://bmjopen.bmj.com/site/about/guidelines.xhtml

For peer review only

24 Barbui C, Cipriani A, Patel V, *et al.* Efficacy of antidepressants and benzodiazepines in minor depression: systematic review and meta-analysis. *Br J Psychiatry* 2011;198:11–6, sup 1. doi:10.1192/bjp.bp.109.076448

25 Cuijpers P, Koole SL, van Dijke A, *et al.* Psychotherapy for subclinical depression: meta-analysis. *Br J Psychiatry* 2014;205:268–74. doi:10.1192/bjp.bp.113.138784

26 Cuijpers P, Koole SL, van Dijke A, *et al.* Psychotherapy for subclinical depression: meta-analysis. *Br J Psychiatry* 2014;205:268–74. doi:10.1192/bjp.bp.113.138784

27 Brookes ST, Whitely E, Egger M, *et al.* Subgroup analyses in randomized trials: risks of subgroup-specific analyses; power and sample size for the interaction test. *J Clin Epidemiol* 2004;57:229–36. doi:10.1016/j.jclinepi.2003.08.009

28 Clarke MJ. Individual patient data meta-analyses. *Best Pract Res Clin Obstet Gynaecol* 2005;19:47–55. doi:10.1016/j.bpobgyn.2004.10.011

29 Jones AP, Riley RD, Williamson PR, *et al.* Meta-analysis of individual patient data versus aggregate data from longitudinal clinical trials. *Clin Trials* 2009;6:16–27. doi:10.1177/1740774508100984

30 Riley RD, Lambert PC, Abo-Zaid G. Meta-analysis of individual participant data: rationale, conduct, and reporting. *BMJ* 2010;340:c221. doi:10.1136/bmj.c221

31 Cooper H, Patall EA. The relative benefits of meta-analysis conducted with individual participant data versus aggregated data. *Psychol Methods* 2009;14:165–76. doi:10.1037/a0015565

32 Campbell LF, Norcross JC, Vasquez MJT, *et al.* Recognition of psychotherapy effectiveness: the APA resolution. *Psychotherapy* 2013;50:98.

33 Norcross JC. An eclectic definition of psychotherapy. *What is Psychother* 1990;:218–20.

34 Lobbestael J, Leurgans M, Arntz A. Inter-rater reliability of the Structured Clinical Interview for DSM-IV Axis I Disorders (SCID I) and Axis II Disorders (SCID II). *Clin Psychol Psychother* 2011;18:75–9. doi:10.1002/cpp.693

35 Robins LN, Wing J, Wittchen HU, *et al.* The Composite International Diagnostic Interview. An epidemiologic Instrument suitable for use in conjunction with different diagnostic systems and in different cultures. *Arch Gen Psychiatry* 1988;45:1069–77.http://www.ncbi.nlm.nih.gov/pubmed/2848472 (accessed 9 Apr 2017).

For peer review only

For peer review only - http://bmjopen.bmj.com/site/about/guidelines.xhtml

36 Sheehan D V, Lecrubier Y, Sheehan KH, *et al.* The Mini-International Neuropsychiatric Interview (M.I.N.I.): the development and validation of a structured diagnostic psychiatric interview for DSM-IV and ICD-10. *J Clin Psychiatry* 1998;:22-33-57.http://www.ncbi.nlm.nih.gov/pubmed/9881538 (accessed 9 Apr 2017).

37 Beck AT, Steer A, Brown GK. *BDI-II: Beck Depression Inventory Manual. 2nd ed. San Antonio, TX: Psychological Corporation; 1996*. 2nd ed. San Antonio: : Psychological Corporation 1996.

38 HAMILTON M. A rating scale for depression. *J Neurol Neurosurg Psychiatry* 1960;23:56–62.

39 Radloff LS. The CES-D Scale: A Self-Report Depression Scale for Research in the General Population. *Appl Psychol Meas* 1977;1:385–401. doi:10.1177/014662167700100306

40 Wahl I, Löwe B, Bjorner JB, *et al.* Standardization of depression measurement: a common metric was developed for 11 self-report depression measures. *J Clin Epidemiol* 2014;67:73–86. doi:10.1016/j.jclinepi.2013.04.019

41 Jacobson NS, Truax P. Clinical significance: a statistical approach to defining meaningful change in psychotherapy research. *J Consult Clin Psychol* 1991;59:12–9.

42 Dolan P. Modeling valuations for EuroQol health states. *Med Care* 1997;35:1095–108.

43 Brazier JE, Rowen D, Hanmer J. Revised SF-6D scoring programmes: a summary of improvements. *PRO Newsl* 2008;40:14–5.

44 Bjelland I, Dahl AA, Haug TT, *et al.* The validity of the Hospital Anxiety and Depression Scale. An updated literature review. *J Psychosom Res* 2002;52:69–77.

45 de Lima Osório F, Crippa JAS, Loureiro SR. Further psychometric study of the Beck Anxiety Inventory including factorial analysis and social anxiety disorder screening. *Int J Psychiatry Clin Pract* 2011;15:255–62. doi:10.3109/13651501.2011.605955

46 Kessler RC, Van Loo HM, Wardenaar KJ, *et al.* Using patient self-reports to study heterogeneity of treatment effects in major depressive disorder. *Epidemiol Psychiatr Sci* 2017;26. doi:10.1017/S2045796016000020

47 Kessler RC, Van Loo HM, Wardenaar KJ, *et al.* Testing a machine-learning algorithm to predict the persistence and severity of major depressive disorder from baseline self-reports. *Mol Psychiatry* 2016;21:366–71. doi:10.1038/mp.2015.198

48 Nigatu YT, Liu Y, Wang J. External validation of the international risk prediction algorithm for major depressive episode in the US general population: the PredictD-US study. *BMC*

For peer review only - http://bmjopen.bmj.com/site/about/guidelines.xhtml

Psychiatry 2016;16:256. doi:10.1186/s12888-016-0971-x

49 King M, Bottomley C, Bellón-Saameño JA, *et al.* An international risk prediction algorithm for the onset of generalized anxiety and panic syndromes in general practice attendees: predictA. *Psychol Med* 2011;41:1625–39. doi:10.1017/S0033291710002400

50 Liu Y, Sareen J, Bolton J, *et al.* Development and validation of a risk-prediction algorithm for the recurrence of panic disorder. *Depress Anxiety* 2015;32:341–8. doi:10.1002/da.22359

51 Støen Grotmol K, Gude T, Moum T, *et al.* Risk factors at medical school for later severe depression: A 15-year longitudinal, nationwide study (NORDOC). *J Affect Disord* 2013;146:106–11. doi:10.1016/j.jad.2012.08.047

52 Bromberger JT, Schott L, Kravitz HM, *et al.* Risk factors for major depression during midlife among a community sample of women with and without prior major depression: are they the same or different? *Psychol Med* 2015;45:1653–64. doi:10.1017/S0033291714002773

53 Hölzel L, Härter M, Reese C, *et al.* Risk factors for chronic depression — A systematic review. *J Affect Disord* 2011;129:1–13. doi:10.1016/j.jad.2010.03.025

54 Lewinsohn PM, Gotlieb IH, Seeley JR. Adolescent Psychopathology: IV. Specificity of Psychosocial Risk Factors for Depression and Substance Abuse in Older Adolescents. *J Am Acad Child Adolesc Psychiatry* 1995;34:1221–9. doi:10.1097/00004583-199509000-00021

55 Kounali D, Zammit S, Wiles N, *et al.* Common versus psychopathology-specific risk factors for psychotic experiences and depression during adolescence. *Psychol Med* 2014;44:2557–66. doi:10.1017/S0033291714000026

56 Heslin M, Desai R, Lappin JM, *et al.* Biological and psychosocial risk factors for psychotic major depression. *Soc Psychiatry Psychiatr Epidemiol* 2016;51:233–45. doi:10.1007/s00127-015-1131-1

57 Chang S-C, Pan A, Kawachi I, *et al.* Risk factors for late-life depression: A prospective cohort study among older women. *Prev Med (Baltim)* Published Online First: 2016. doi:10.1016/j.ypmed.2016.08.014

58 Chan MF, Zeng W. Exploring risk factors for depression among older men residing in Macau. *J Clin Nurs* 2011;20:2645–54. doi:10.1111/j.1365-2702.2010.03689.x

59 Zhou X, Bi B, Zheng L, *et al.* The prevalence and risk factors for depression symptoms in a rural Chinese sample population. *PLoS One* 2014;9:e99692. doi:10.1371/journal.pone.0099692

For peer review only - http://bmjopen.bmj.com/site/about/guidelines.xhtml

For peer review only

60 Whiteman K, Ruggiano N, Thomlison B. Transforming mental health services to address gender disparities in depression risk factors. *J Women Aging* 2016;:1–9. doi:10.1080/08952841.2015.1072027

61 Tang B, Liu X, Liu Y, *et al.* A meta-analysis of risk factors for depression in adults and children after natural disasters. *BMC Public Health* 2014;14:623. doi:10.1186/1471-2458-14-623

62 Nakulan A, Sumesh TP, Kumar S, *et al.* Prevalence and risk factors for depression among community resident older people in Kerala. *Indian J Psychiatry* 2015;57:262–6. doi:10.4103/0019-5545.166640

63 Salokangas RKR, Poutanen O. Risk factors for depression in primary care: findings of the TADEP project. *J Affect Disord* 1998;48:171–80. doi:10.1016/S0165-0327(97)00171-7

64 Li J, Theng Y-L, Foo S. Depression and Psychosocial Risk Factors among Community-Dwelling Older Adults in Singapore. *J Cross Cult Gerontol* 2015;30:409–22. doi:10.1007/s10823-015-9272-y

65 Pelkonen M, Marttunen M, Kaprio J, *et al.* Adolescent risk factors for episodic and persistent depression in adulthood. A 16-year prospective follow-up study of adolescents. *J Affect Disord* 2008;106:123–31. doi:10.1016/j.jad.2007.06.001

66 MacPhee AR, Andrews JJW. Risk factors for depression in early adolescence. *Adolescence* 2006;41:435–66.

67 Czeglédi E, Urbán R. [Risk factors and alteration of depression among participants of an inpatient weight loss program]. *Psychiatr Hungarica A Magy Pszichiátriai Társaság tudományos folyóirata* 2012;27:361–78.

68 Yanzón de la Torre A, Oliva N, Echevarrieta PL, *et al.* Major depression in hospitalized Argentine general medical patients: Prevalence and risk factors. *J Affect Disord* 2016;197:36–42. doi:10.1016/j.jad.2016.02.066

69 Brausch AM, Gutierrez PM. The role of body image and disordered eating as risk factors for depression and suicidal ideation in adolescents. *Suicide Life Threat Behav* 2009;39:58–71. doi:10.1521/suli.2009.39.1.58

70 Hoare E, Skouteris H, Fuller-Tyszkiewicz M, *et al.* Associations between obesogenic risk factors and depression among adolescents: a systematic review. *Obes Rev* 2014;15:40–51. doi:10.1111/obr.12069

71 Wild J, Smith K V, Thompson E, *et al.* A prospective study of pre-trauma risk factors for post-

For peer review only

traumatic stress disorder and depression. *Psychol Med* 2016;46:2571–82. doi:10.1017/S0033291716000532

72 Miletic V, Lukovic JA, Ratkovic N, *et al.* Demographic risk factors for suicide and depression among Serbian medical school students. *Soc Psychiatry Psychiatr Epidemiol* 2015;50:633–8. doi:10.1007/s00127-014-0950-9

73 Sajjadi H, Mohaqeqi Kamal SH, Rafiey H, *et al.* A systematic review of the prevalence and risk factors of depression among iranian adolescents. *Glob J Health Sci* 2013;5:16–27. doi:10.5539/gjhs.v5n3p16

74 Anstey KJ, von Sanden C, Sargent-Cox K, *et al.* Prevalence and risk factors for depression in a longitudinal, population-based study including individuals in the community and residential care. *Am J Geriatr Psychiatry* 2007;15:497–505. doi:10.1097/JGP.0b013e31802e21d8

75 Lu W, Bian Q, Song Y, *et al.* Prevalence and related risk factors of anxiety and depression among Chinese college freshmen. *J Huazhong Univ Sci Technolog Med Sci* 2015;35:815–22. doi:10.1007/s11596-015-1512-4

76 Cuijpers P, Huibers M, Ebert DD, *et al.* How much psychotherapy is needed to treat depression? A metaregression analysis. *J Affect Disord* 2013;149:1–13. doi:10.1016/j.jad.2013.02.030

77 Cuijpers P, van Straten A, Warmerdam L, *et al.* Psychological treatment of depression: a meta-analytic database of randomized studies. *BMC Psychiatry* 2008;8:36. doi:10.1186/1471-244x-8-36

78 Higgins JM, Altman DG. Assessing Risk of Bias in included studies. In: *Cochrane Handbook for Systematic Reviews of Interventions:* John Wiley & Sons, Ltd. pp 2008. 187–241.

79 Quartagno M, Carpenter JR. Multiple imputation for IPD meta-analysis: allowing for heterogeneity and studies with missing covariates. *Stat Med* 2016;35:2938–54. doi:10.1002/sim.6837

80 Lüdtke O, Robitzsch A, Grund S. Multiple imputation of missing data in multilevel designs: A comparison of different strategies. *Psychol Methods* 2017;22:141–65. doi:10.1037/met0000096

81 Enders CK, Mistler SA, Keller BT. Multilevel multiple imputation: A review and evaluation of joint modeling and chained equations imputation. *Psychol Methods* 2016;21:222–40. doi:10.1037/met0000063

For peer review only - http://bmjopen.bmj.com/site/about/guidelines.xhtml

82 Quartagno M, Maintainer JC. R Package ‘jomo’ Multilevel Joint Modelling Multiple Imputation. Published Online First: 2016.https://cran.r-project.org/web/packages/jomo/jomo.pdf (accessed 9 Apr 2017).

83 Grund S, Lüdtke O, Robitzsch A. Multiple Imputation of Multilevel Missing Data. *SAGE Open* 2016;6:215824401666822. doi:10.1177/2158244016668220

84 DerSimonian R, Laird N. Meta-analysis in clinical trials. *Control Clin Trials* 1986;7:177–88.

85 Orsini N, Higgins J, Bottai M, *et al.* Heterogi: Stata module to quantify heterogeneity in a Meta-analysis. 2013.

86 Duval S, Tweedie R. Trim and fill: A simple funnel-plot-based method of testing and adjusting for publication bias in meta-analysis. *Biometrics* 2000;56:455–63.

87 Borenstein M, Hedges L V., Higgins JPT, *et al. Introduction to Meta-Analysis*. Chichester, UK: : John Wiley & Sons, Ltd 2009. doi:10.1002/9780470743386

88 Mathew T, Nordström K. Comparison of one-step and two-step meta-analysis models using individual patient data. *Biom J* 2010;52:271–87. doi:10.1002/bimj.200900143

89 Martuzzi M, Elliott P. Estimating the incidence rate ratio in cross-sectional studies using a simple alternative to logistic regression. *Ann Epidemiol* 1998;8:52–5.http://www.ncbi.nlm.nih.gov/pubmed/9465994 (accessed 16 May 2017).

90 Rush AJ, Kraemer HC, Sackeim HA, *et al.* Report by the ACNP Task Force on response and remission in major depressive disorder. *Neuropsychopharmacology* 2006;31:1841–53. doi:10.1038/sj.npp.1301131

91 Laupacis A, Sackett DL, Roberts RS. An assessment of clinically useful measures of the consequences of treatment. *N Engl J Med* 1988;318:1728–33. doi:10.1056/NEJM198806303182605

92 Ebert DD, Donkin L, Andersson G, *et al.* Does Internet-based guided-self-help for depression cause harm? An individual participant data meta-analysis on deterioration rates and its moderators in randomized controlled trials. *Psychol Med* 2016;46. doi:10.1017/S0033291716001562

93 Bower P, Kontopantelis E, Sutton A, *et al.* Influence of initial severity of depression on effectiveness of low intensity interventions: meta-analysis of individual patient data. *BMJ* 2013;346:f540.

For peer review only

For peer review only - http://bmjopen.bmj.com/site/about/guidelines.xhtml

94 Karyotaki E, Riper H, Twisk J, *et al.* Efficacy of Self-guided Internet-Based Cognitive Behavioral Therapy in the Treatment of Depressive Symptoms. *JAMA Psychiatry* Published Online First: 22 February 2017. doi:10.1001/jamapsychiatry.2017.0044

95 Karyotaki E, Kleiboer A, Smit F, *et al.* Predictors of treatment dropout in self-guided web-based interventions for depression: an 'individual patient data' meta-analysis. *Psychol Med* 2015;45:2717–26. doi:10.1017/S0033291715000665

96 Cuijpers P, Weitz E, Twisk J, *et al.* Gender as predictor and moderator of outcome in cognitive behavior therapy and pharmacotherapy for adult depression: an "individual patient data" meta-analysis. *Depress Anxiety* 2014;31:941–51. doi:10.1002/da.22328

97 Weitz ES, Hollon SD, Twisk J, *et al.* Baseline Depression Severity as Moderator of Depression Outcomes Between Cognitive Behavioral Therapy vs Pharmacotherapy: An Individual Patient Data Meta-analysis. *JAMA psychiatry* 2015;72:1102–9. doi:10.1001/jamapsychiatry.2015.1516

98 Bennett K, Manassis K, Walter SD, *et al.* COGNITIVE BEHAVIORAL THERAPY AGE EFFECTS IN CHILD AND ADOLESCENT ANXIETY: AN INDIVIDUAL PATIENT DATA METAANALYSIS. *Depress Anxiety* 2013;30:829–41. doi:10.1002/da.22099

99 Furukawa TA, Schramm E, Weitz ES, *et al.* Cognitive-Behavioural Analysis System of Psychotherapy (CBASP), a drug, or their combination: differential therapeutics for persistent depressive disorder: a study protocol of an individual participant data network meta-analysis. *BMJ Open* 2016;6:e011769. doi:10.1136/bmjopen-2016-011769

100 Purgato M, Gross AL, Jordans MJ, *et al.* Psychosocial interventions for children exposed to traumatic events in low- and middle-income countries: study protocol of an individual patient data meta-analysis. *Syst Rev* 2014;3:34. doi:10.1186/2046-4053-3-34

101 Weitz E, Kleiboer A, van Straten A, *et al.* Individual patient data meta-analysis of combined treatments versus psychotherapy (with or without pill placebo), pharmacotherapy or pill placebo for adult depression: a protocol. *BMJ Open* 2017;7:e013478. doi:10.1136/bmjopen-2016-013478

102 Jolani S, Debray TPA, Koffijberg H, *et al.* Imputation of systematically missing predictors in an individual participant data meta-analysis: a generalized approach using MICE. *Stat Med* 2015;34:1841–63. doi:10.1002/sim.6451

For peer review only

For peer review only - http://bmjopen.bmj.com/site/about/guidelines.xhtml

PRISMA-P (Preferred Reporting Items for Systematic review and Meta-Analysis Protocols) 2015 checklist: recommended items to address in a systematic review protocol*

Section and topic	Item No	Checklist item	page
**ADMINISTRATIVE INFORMATION**			
Title:			
Identification	1a	Identify the report as a protocol of a systematic review	1
Update	1b	If the protocol is for an update of a previous systematic review, identify as such	/
Registration	2	If registered, provide the name of the registry (such as PROSPERO) and registration number	2
Authors:			
Contact	3a	Provide name, institutional affiliation, e-mail address of all protocol authors; provide physical mailing address of corresponding author	1
Contributions	3b	Describe contributions of protocol authors and identify the guarantor of the review	14
Amendments	4	If the protocol represents an amendment of a previously completed or published protocol, identify as such and list changes; otherwise, state plan for documenting important protocol amendments	/
Support:			
Sources	5a	Indicate sources of financial or other support for the review	14
Sponsor	5b	Provide name for the review funder and/or sponsor	14
Role of sponsor or funder	5c	Describe roles of funder(s), sponsor(s), and/or institution(s), if any, in developing the protocol	14
**INTRODUCTION**			
Rationale	6	Describe the rationale for the review in the context of what is already known	4
Objectives	7	Provide an explicit statement of the question(s) the review will address with reference to participants, interventions, comparators, and outcomes (PICO)	5
**METHODS**			
Eligibility criteria	8	Specify the study characteristics (such as PICO, study design, setting, time frame) and report characteristics (such as years considered, language, publication status) to be used as criteria for eligibility for the review	5,6
Information sources	9	Describe all intended information sources (such as electronic databases, contact with study authors, trial registers or other grey literature sources) with planned dates of coverage	7,8
Search strategy	10	Present draft of search strategy to be used for at least one electronic database, including planned limits, such that it could be repeated	7
Study records:			
Data	11a	Describe the mechanism(s) that will be used to manage records and data throughout the review	8

For peer review only

management			
Selection process	11b	State the process that will be used for selecting studies (such as two independent reviewers) through each phase of the review (that is, screening, eligibility and inclusion in meta-analysis)	7,8
Data collection process	11c	Describe planned method of extracting data from reports (such as piloting forms, done independently, in duplicate), any processes for obtaining and confirming data from investigators	8
Data items	12	List and define all variables for which data will be sought (such as PICO items, funding sources), any pre-planned data assumptions and simplifications	6,7
Outcomes and prioritization	13	List and define all outcomes for which data will be sought, including prioritization of main and additional outcomes, with rationale	6
Risk of bias in individual studies	14	Describe anticipated methods for assessing risk of bias of individual studies, including whether this will be done at the outcome or study level, or both; state how this information will be used in data synthesis	8
Data synthesis	15a	Describe criteria under which study data will be quantitatively synthesised	9
	15b	If data are appropriate for quantitative synthesis, describe planned summary measures, methods of handling data and methods of combining data from studies, including any planned exploration of consistency (such as I^2, Kendall's τ)	6,9
	15c	Describe any proposed additional analyses (such as sensitivity or subgroup analyses, meta-regression)	11
	15d	If quantitative synthesis is not appropriate, describe the type of summary planned	/
Meta-bias(es)	16	Specify any planned assessment of meta-bias(es) (such as publication bias across studies, selective reporting within studies)	9
Confidence in cumulative evidence	17	Describe how the strength of the body of evidence will be assessed (such as GRADE)	12,13

*** It is strongly recommended that this checklist be read in conjunction with the PRISMA-P Explanation and Elaboration (cite when available) for important clarification on the items. Amendments to a review protocol should be tracked and dated.** **The copyright for PRISMA-P (including checklist) is held by the PRISMA-P Group and is distributed under a Creative Commons Attribution Licence 4.0.**

From: Shamseer L, Moher D, Clarke M, Ghersi D, Liberati A, Petticrew M, Shekelle P, Stewart L, PRISMA-P Group. Preferred reporting items for systematic review and meta-analysis protocols (PRISMA-P) 2015: elaboration and explanation. BMJ. 2015 Jan 2;349(jan02 1):g7647.

For peer review only
